# *Dipsacus* and *Scabiosa* Species—The Source of Specialized Metabolites with High Biological Relevance: A Review

**DOI:** 10.3390/molecules28093754

**Published:** 2023-04-27

**Authors:** Ewa Skała, Agnieszka Szopa

**Affiliations:** 1 Department of Biology and Pharmaceutical Botany, Medical University of Lodz, Muszynskiego 1, 90-151 Lodz, Poland; 2 Chair and Department of Pharmaceutical Botany, Medical College, Jagiellonian University, Medyczna 9, 30-688 Kraków, Poland

**Keywords:** *Dipsaci radix*, xu duan, *D. fullonum*, *D. inermis*, *D. japonicus*, *Scabiosa atropurpurea*, *S. comosa*, Qingganjiuwei, Gurigumu-7, Xian-Ling-Gu-Bao

## Abstract

The genera *Dipsacus* L. and *Scabiosa* L. of the Caprifoliaceae family are widely distributed in Europe, Asia, and Africa. This work reviews the available literature on the phytochemical profiles, ethnomedicinal uses, and biological activities of the most popular species. These plants are rich sources of many valuable specialized metabolites with beneficial medicinal properties, such as triterpenoid derivatives, iridoids, phenolic acids, and flavonoids. They are also sources of essential oils. The genus *Dipsacus* has been used for centuries in Chinese and Korean folk medicines to treat bone (osteoporosis) and joint problems (rheumatic arthritis). The Korean Herbal Pharmacopoeia and Chinese Pharmacopoeia include *Dipsaci radix*, the dried roots of *D. asperoides* C.Y.Cheng & T.M.Ai. In addition, *S. comosa* Fisch. ex Roem & Schult. and *S. tschiliiensis* Grunning are used in traditional Mongolian medicine to treat liver diseases. The current scientific literature data indicate that these plants and their constituents have various biological properties, including inter alia antiarthritic, anti-neurodegenerative, anti-inflammatory, antioxidant, anticancer, and antimicrobial activities; they have also been found to strengthen tendon and bone tissue and protect the liver, heart, and kidney. The essential oils possess antibacterial, antifungal, and insecticidal properties. This paper reviews the key biological values of *Dipsacus* and *Scabiosa* species, as identified by in vitro and in vivo studies, and presents their potential pharmacological applications.

## 1. Introduction

Fruits, vegetables, and herbs provide the body with many valuable specialized metabolites, often with pro-health properties. These include polyphenols, alkaloids, terpenoids, and essential oils, all of which display specific biological activities. Plants have been utilized in folk medicine in many countries for centuries because of their long-known therapeutic benefits. Not only have the beneficial medicinal properties of plants have been known for thousands of years, consumer interest in phytotherapy/herbal medicines and natural food supplements continues to grow, and in many countries, traditional medicine is the only mode of treatment for many diseases.

Although herbal materials have many ethnomedicinal benefits, their toxicity or potential side effects remain relatively unexplored, and their medical potential frequently lacks a scientific basis. By determining the chemical composition of plant extracts, it is possible to estimate their safety and biological activities and hence their potential as natural drugs. The use of medicinal plants as natural sources of compounds with inter alia antioxidant, anti-inflammatory, and anti-diabetic properties has drawn the attention of many researchers. Oxidative stress and the inflammatory response are associated with many neurological diseases (Alzheimer’s disease, Parkinson’s disease, amyotrophic lateral sclerosis), cardiovascular diseases (atherosclerosis), hepatic conditions, gastrointestinal diseases, and cancers [1,2,3].

Recent years have seen a greater interest in the genera *Dipsacus* L. (teasel in English) and *Scabiosa* L. (pincushions in English) [4], with the latter being poorly understood. The first mention of phytochemical studies on *Dipsacus* spp. dates to the 1920s [5]. In 2011, Zhao and Shi [5] reviewed the chemical composition and biological properties of the specialized metabolites of some *Dipsacus* species. Ten years later, Tao et al. [6] published a review of the literature regarding one *Dipsacus* species, *D. asper* Wall. ex C.B.Clarke, its chemical constituents, selected pharmacological activities, and pharmacokinetics. In 2018, Pinto et al. [7] provided a review of flavonoids and terpenoid derivatives identified in some species from *Scabiosa* and their biological effects. In recent years, a significant number of reports have been published on the chemical composition and biological properties of the two genera.

Therefore, the present review aims to provide an overview of the current literature (until December 2022) regarding the phytochemistry, biological activities, and toxicology of selected species of *Dipsacus* and *Scabiosa*. Several online databases, including PubMed, Google Scholar, Scopus, and ScienceDirect, were searched in the current review. These two genera include many synonymous Latin species names, meaning the same species, which can lead to confusion. In the present review, the species names are cited according to the authors of publications. The natural occurrence of *Dipsacus* and *Scabiosa* species as well as synonymous species names are described based on the data from The World Flora Online, Plants of the World Online, The Global Biodiversity Information Facility, and Flora of China [4,8,9,10] (Table 1).

The genera *Dipsacus* and *Scabiosa* currently belong to the Caprifoliaceae Juss. family (honeysuckle family) [7,8]. They were previously classified taxonomically in the Dipsaceae [7]. According to Plants of the World Online database [9], the Caprifoliaceae includes 33 accepted genera. This family has been divided into six subfamilies and one genus. Both genera *Dipsacus* and *Scabiosa* are classified into the subfamily Dipsacoideae and order Dipsacales [8,54]. The genus *Dipsacus* is widely distributed in Europe, North Africa, and Asia in North Myanmar and comprises 21 accepted species [9]. The native range of the genus *Scabiosa* includes Eurasia, Macaronesia to North Africa, Eritrea, and South Africa, with 66 accepted species [9]. In particular, numerous representatives of European *Scabiosa* species appear in the Mediterranean region [55,56]. Table S1 (Supplementary Material) presents accepted species of the genera *Dipsacus* and *Scabiosa*. *Dipsacus* includes various ornamental plants used in floristry for their decorative dried inflorescences. The dried inflorescences were previously used in the textile industry to clean and lift the nap on woolen fabrics [57,58]. The name *dipsacus* itself is believed to be derived from the Greek word for *dipsa* or thirst [9]. The genus name *scabiosa* is derived from the Latin word *scabies* or itch [9]. According to Akar [59], *Scabiosa* plants were traditionally used to treat scabies, skin sores, and other skin infections.

## 2. Traditional Medicinal Uses and Pharmacopoeial Monographs

Many *Dipsacus* and *Scabiosa* species are known in various traditional medicines, including traditional Chinese medicine. So far, among over 20 species of the genus *Dipsacus* and 60 species of *Scabiosa*, the phytochemical profiles and biological properties of only a few species are known. As such, there is a need to better understand the chemical composition of these genera and their potential medicinal value.

### 2.1. Dipsacus spp.

Numerous, recent reports have examined the potential therapeutic effects of *Dipsacus* genera. However, most phytochemical and biological studies have focused on the biological activities and health-promoting factors of *D. asperoides* C.Y.Cheng et T.M.Ai (=*D. asper* Wall. ex C.B.Clarke) [6]. The oldest report on *Dipsacus* genus can be found in *Shen Nong’s Herbal Classic* (*Shen Nong Ben Cao Jing* in Chinese) [6,11,18]. *D. asper* is widespread in the southern and northern regions of China such as the Hunan, Yunnan, Gansu, and Shanxi Provinces [12,19]. The growing demand for this species and its mass harvesting has significantly weakened its population in a natural state [19,60]. *D. asperoides* is cultivated on a large scale, mainly in Hefeng City, Hubei Province. This species is also cultivated in other Chinese cities, including Xichang, Sichuan, Xifeng, Guizhou, Jianchuan, and Yunnan Provinces as well as Jiangxi and Guangxi Provinces in China [18,60].

The roots of *D. asperoides* in China are commonly known as Xu Duan or Himalayan Teasel Roots [6,11,12,13,14,15,16,17,18,20,61]. Traditionally, in China and Korea, *Dipsaci radix* is known as the raw material used in treating joint disease (rheumatic arthritis) and bone diseases (osteoporosis, bone fractures), lumbar and knee pain, arthralgia, traumatic hematoma, uterine bleeding, and gynecological diseases; it is also used to strengthen muscles and improve liver and kidney functions [6,12,15,26,27,28,62,63,64]. The raw material is collected in autumn. *Dipsaci radix* is 5–15 cm in length and 0.5–2 cm in diameter, with slightly twisted or twisted longitudinal wrinkles and furrows. It is greyish-brown or yellowish-brown in color [28]. *Dipsaci radix* has a spicy, bitter, slightly sweet, then astringent taste [26,28,29].

*Dipsaci radix* can be subjected to diaphoretic-, salt-, and wine-processing methods [6]. Some researchers suggest that the procedure of herb processing may result in differences in specialized metabolite content and biological effects. Some studies indicate that wine processing yields higher levels of key compounds (e.g., asperosaponin V and VI, dipsacoside A and B, dipsacussaponin B, loganic acid, and chlorogenic acid) than crude *Dipsaci radix* [65,66]. Materials processed by rice wine may promote anti-osteoporosis, anti-inflammatory, anti-coagulant, and analgesic activities or have a beneficial effect on blood circulation [6,67,68].

*Dipsaci radix* is commonly available in the Chinese herbal medicine market. This herb is mainly produced in China, in the Provinces of Sichuan, Yunnan, Hubei, Hunan, Xizang, Jiangxi, Guizhou, and Guangxi [18,20]. *Dipsaci radix* has a pharmacopeial monograph in the Korean Herbal Pharmacopoeia (Korea Food and Drug Administration 2007) and the 10th Chinese Pharmacopoeia, 2015 edition. The raw material is standardized to contain akebia saponin D whose level should be above 2% [69]. Chun et al. [30] indicate that the traditional use of *D. asperoides* is associated with its analgesic and anti-inflammatory properties for treating inter alia rheumatoid arthritis and bone fractures. The Chinese Pharmacopoeia, 2015 edition, recommends 9–15 g as the daily dose of *D. asper* for humans [15].

In the Korean herbal medicine markets, *Phlomidis radix* from *Phlomis umbrosa* Turczaninow is often sold instead of *Dipsaci radix* or both raw materials are mixed [70]. This mistake results from the morphological similarity of these two dried raw materials and their names [69,70]. Both species have been used in the Korean medicine Sok-dan for the treatment of bone- and arthritis-related diseases and are listed in the Korean Herbal Pharmacopoeia [70].

HPLC/UV profiles of four samples of *Phlomidis radix* and 17 samples of *Dipsaci radix* demonstrated that loganin, sweroside, dipsanoside A, 3-*O*-[*β*-d-glu-(1→4)][α-l-rha-(1→3)]-*β*-d-glu(1→3)-α-l-rha-(1→2)-α-l-ara-hed 28-*O*-*β*-d-glu-(1→6)-β-d-glu ester, and akebia saponin D were not detected in *Phlomidis radix*. The levels of these compounds in *Dipsaci radix* varied depending on the origin of the material and the extraction methods [69]. Akebia saponin D (a quality indicator of *Dipsaci radix*) predominated, with a content range of 0.73–10.96% (*w*/*w*) [69]. Both herbs showed anti-osteoarthritis ability in a Sprague-Dawley rat model induced by monosodium iodoacetate intra-articular knee injections [70]. However, *Phlomidis radix* was found to be more effective, suggesting that it may be used as an alternative to *Dipsaci radix*. It was found that the 70% ethanol extract (200 mg/kg/day) administered as oral gavage for 21 days restored the weight-bearing ability of the hind paw, suppressed histopathological changes of the osteoarthritic knee, inhibited the serum levels of inflammation mediators tumor necrosis factor α (TNF-α) and interleukin-1β (IL-1β), and inhibited the over-expression of the gene encoding matrix-degrading metalloproteinases MMP-9 and MMP-13 in the knee joint tissue [70]. In addition, the ethanol extract of *Dipsaci radix* displayed a protective effect against the destruction of articular cartilage, elevated myeloperoxidase (MPO) and down-regulated dystonin gene expression, modulated WNT/β-catenin signaling pathway, suppressed gene expression of Adamts4, and increased the expression of cartilage collagen genes (e.g., Col2A1, Col9A1*,* and Col11A1) and SOX5, SOX9, and Frzb genes [30].

Another species of *Dipsacus* is *D. fullonum* L., commonly known as teasel or wild teasel [33,34]. Traditionally, *D. fullonum* has been used to treat Lyme disease [35] and eye infections in cattle [36]. *D. fullonum* is distributed naturally in Europe to the Caucasus and North-Western Africa [9]. A rich source of valuable ingredients used in the traditional medicine of the Kashmir Himalayas is *D. inermis* Wall., also known as Wopal haakh/Wopal Hak in the Kashmiri language. It is used in treating cold, fever, cough, sore throat, general fatigue, and body pain and has demonstrated stomachic and carminative properties [37,38,39,71]. The roots of *D. japonicus* Miq., commonly known as Tuc doan in Vietnam, show tonic, anodyne, and demulcent activities. The recommended daily dose is 10–20 g as a decoction, alcoholic maceration, powder, or pill [41]. Decoction of *D. japonicus* roots has been used in the traditional medicine of China and Vietnam for rheumatism, sprains, trauma, fractures, relieving joint pain and ostealgia, and hepatic and renal hypofunction [40,41]. *D. japonicus* is widespread in central and northern China, Korea, Japan, and Vietnam [9,40]. The leaves of *D. sativus* (L.) Honck. are used as an infusion for the treatment of cardiovascular diseases. This species was originally cultured in Europe and was introduced from Japan to China in 1929 [42].

### 2.2. Scabiosa spp.

With respect to *Scabiosa* genera, many studies indicate that species of this genus show antioxidant [42,43,46,50,52,53,56,72,73,74,75,76,77], anti-inflammatory [50], anti-diabetic [43,78], anti-hepatic fibrosis [47,79,80], anti-cancer [56,81], and antibacterial [56,75,77,82,83] properties. Many species of *Scabiosa* grow naturally in the Mediterranean region [55,56].

In Tunisia, species from *Scabiosa* were commonly applied for skin treatment [56]. In the Iberian Peninsula, an infusion of *S. atropurpurea* L. inflorescence is used externally on the skin as an anti-acne treatment and orally for measles, rubeola, and scarlet fever [44,84]. In northern Peru, the aerial parts have been used for menstrual regulation and in Iberia, as a veterinary diuretic [46]. *S. atropurpurea* is distributed throughout the Mediterranean, Europe, Asia, and southern Africa [46]. Genetic investigations show that *S. atropurpurea* has 97% similarity with *S. tschiliensis* and thus is also called Japanese scabiosa [46]. This species is known as Mor uyuzotu or Şeytanotu in Turkey, Ambarina in Northern Peru, and Escabiosa in the Iberian Peninsula [45]. The leaves and flowers of *S. stellata* L. were used in the traditional medicine of Morocco to treat heel cracks [46,48]. *S. stellata* is an endemic plant in North Africa and is commonly known as starflower pincushions [75]. Another endemic species in North Africa (Algeria, Egypt, Libya, Morocco, and Tunisia) is *S. arenaria* Forssk. [9,72]. In traditional Mongolian medicine, the inflorescences of *S. comosa* Fisch. ex Roem. et Schult. and *S. tschilliensis* Grunning (known as Lanpenhua in Chinese) are used for liver diseases [74,79]. *S. tschiliensis* is widespread in China (in Hebei Province) and the Inner Mongolia autonomy district [51,53] and is locally called Meng Gu Shan Luo Bo [51]. Qingganjiuwei powder (composed of nine herbal materials including *S. comosa*) is commonly used as an anti-fibrosis drug in patients with chronic hepatic disease in Inner Mongolia [85,86]. This drug is accepted by the Inner Mongolia Region Drug Administration [86]. The flowers of *S. comosa* (*Scabiosae flos*) are also an ingredient of Gurigumu-7 used in traditional Mongolian and Tibetan medicine to treat liver diseases. This preparation is in the form of a bitter and astringent powder [87].

## 3. Chemical Constituents of *Dipsacus* and *Scabiosa* Species

**Figure 1:**
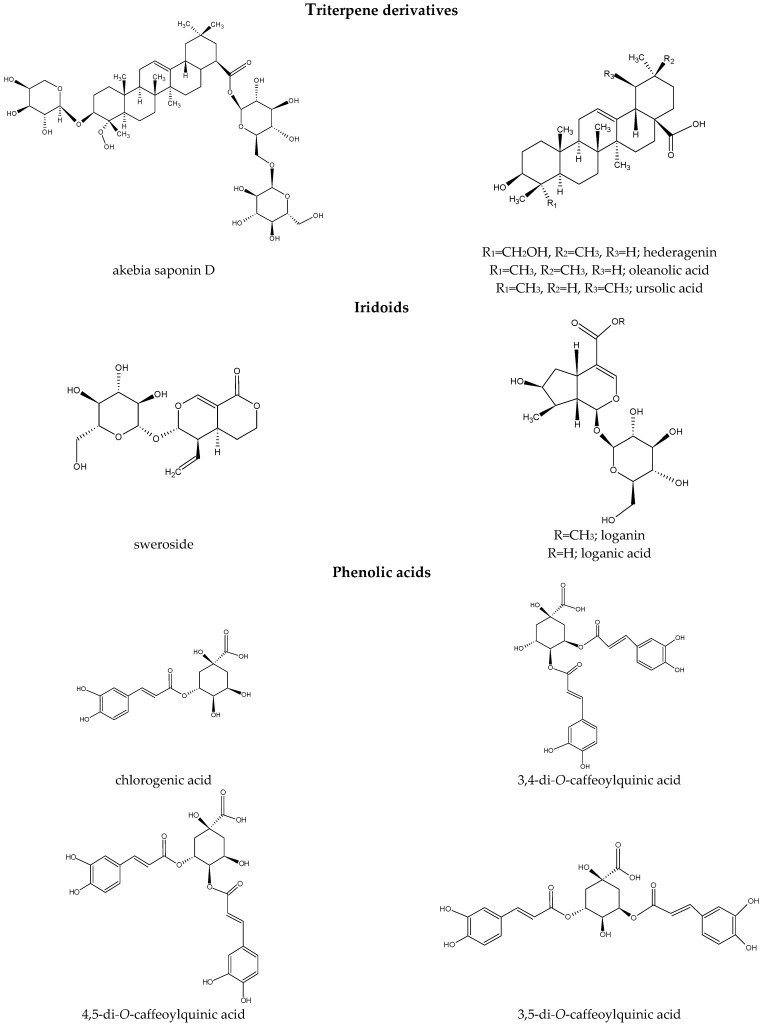

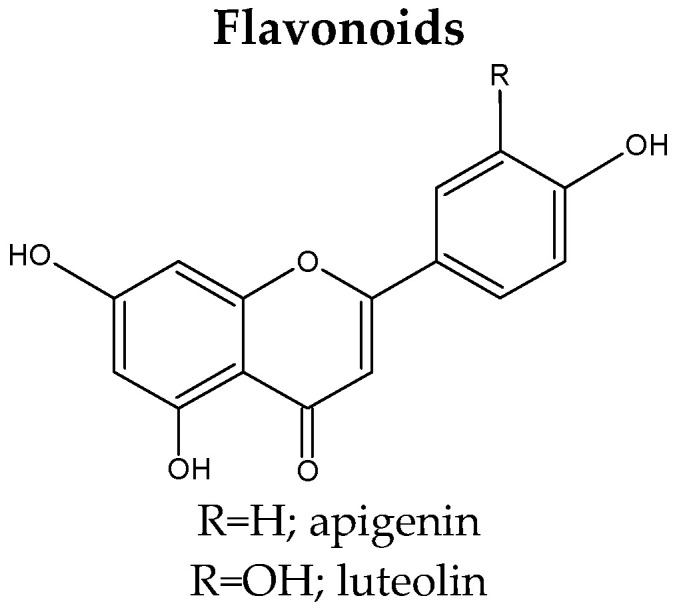
The chemical structures of selected triterpene derivatives, iridoids, phenolic acids, and flavonoids identified in *Dipsacus* and *Scabiosa*. The chemical structures of the secondary metabolites were compiled based on the data reported in [103,104,105,106,107,108].

*Dipsacus* and *Scabiosa* have closely related phytochemical profiles [75] and include over 200 specialized metabolites. Some representatives of *Dipsacus* and *Scabiosa* biosynthesize chemical compounds of various classes, mainly triterpenoid derivatives (Table 2) [5,6,7,12,21,22,27,40,48,49,51,65,66,81,82,88,89,90,91,92,93,94,95,96], which possess a variety of bioactivities. In addition, iridoids [6,7,13,22,27,33,34,65,66,75,78,84,89,92,93,94,95,96,97,98,99,100] (Table 3), phenolic acids [6,24,27,29,33,34,43,46,53,65,66,73,74,75,76,81,89,92,93,94,95,97,99,101] (Table 4), and flavonoids [7,33,34,42,43,46,48,53,73,74,75,76,81,82,94] (Table 5) have also been reported. Further, few alkaloids have been found in *D. asper* such as cantleyine, venoterpine, gentianine, dipsaperine ((3*S*,5*S*)-5-carboxystrictosidic acid 22-loganin ester), 3*β*,5α-tetrahydrodesoxycordifoline lactam, and (3*R*,5*S*)-5-carboxyvincosidic acid 22-loganin ester [6,92,96,98,100], and lignans have been detected [6,13,46,96,97,99] (Table 6). *Scabiosa* species are also rich sources of flavonoids, mainly in the aerial parts and flowers [7,43,46,48,49,53,73,74,75,81,82,94]. Essential oils were also isolated from *S. arenaria*, *S. atropurpurea*, *D. fullonum*, and *D. japonicus* (Table 7) [35,46,83,102]. Fatty acids have also been detected in *Scabiosa* spp. and *D. asper* (Table 8) [6,49,94]. The specialized metabolites identified in *Dipsacus* and *Scabiosa* are listed in Table 2, Table 3, Table 4, Table 5, Table 6, Table 7 and Table 8. The structures of selected compounds that have displayed some biological activities in a number of studies are shown in Figure 1.

### 3.1. Terpenoid Derivatives

The most diverse group of specialized metabolites in *Dipsacus* and *Scabiosa* are the triterpenoid derivatives, which can be divided into oleanane-type, hederagenin-type, or ursane-type (Table 2). Some compounds are derived from pomoic acid (scabiosaponin H-I) [7,51]. In *Dipsacus*, the main group of triterpenoids identified is hederagenin and its related saponins, while the rarest is ursane-type terpenoids [5] (Table 2). The oleanane-type triterpenoids were also common in *Scabiosa* genus (for example, oleanolic acid, scabiosaponins A-G, scabiostellatosides A-F, and hookeroside A and B) (Table 2) [7,48,49,51,82,94]. Some of these specialized metabolites such as oleanolic acid and ursolic acid were detected in the species of both genera [6,7,12,48,49,82,89,94]. Some of them were genus specific, such as hookeroside A and B and scabiosaponin A-K identified in *S. tschiliensis* whole plants [7,51] and palustroside III and scabiostellatosides A-H detected in *S. stellata* whole plants [7,48]. The new, tentatively detected triterpenoid derivatives in *S. atropurpurea* subsp. *maritima* leaves were oleanolic acid-pentosyl-rhamnosyl-pentosyl-glucosyl-di-glucoside, oleanolic acid-pentosyl-rhamnosyl-glucosyl-glucosyl-di-glucoside, and maslinic acid-pentosyl-rhamnosyl-glucosyl-glucosyl [81]. Yu et al. [12,22,96] reported some new arborinane-type triterpenoid (25-acetoxy-28-dehydroxyrubiarbonone E), ursane-type triterpenoids (2*α*,3*β*,24-trihydroxy-23-norurs-12-en-28-oic acid and 2*α*,3*β*-dihydroxy-23-norurs-4(24),11,13(18)-trien-28-oic acid), and oleanane-type triterpenoids (2′,3′-*O*-diacetyl-3-*O*-*α*-l-arabinopyranosyl-23-hydroxyolea-12-en-28-oic acid, 2′,4′-*O*-diacetyl-3-*O*-*α*-l-arabinopyranosyl-23-hydroxyolea-12-en-28-oic acid, and 3-*O*-*β*-d-glucopyranosyl-(1→3)-α-l-rhamnopyranosyl-(1→2)-α-l-arabinopyranosyl-23-hydroxyolean-18-en-28-oic acid 28-*O*-*β*-d-glucopyranosyl-(1→6)-β-d-glucopyranosyl ester) in *D. asper* roots. A new compound elucidated in *D. japonicus* roots was saponin XII [40]. In addition, this species has presented the only example to date of japondipsaponin E1 [5,88].

The indicator of the quality of *Dipsaci radix* is akebia saponin D (asperosaponin VI) [65]. It is known that environmental conditions, geographic location, the growth stage of the plants, or the year of harvest affect the content of the specialized metabolites in plants. Jin et al. [60] demonstrated that the content of akebia saponin D varied in *D. asperoides* roots collected from different geographical regions of China (i.e., Guizhou, Hubei, Sichuan, and Yunnan Provinces). The highest akebia saponin D content (about 6% of dry weight) was noted in roots collected from Hubei. A similar observation was noted by Du et al. [95]. The level of akebia saponin D ranged from 1.61% to 15.19% in samples of different origin.

**Table 2:** Terpenoid derivatives identified in *Dipsacus* and *Scabiosa*.

No.	Compound Name	Identification	Species (Part of Plant)
1.	(6*α*,11*α*)-6-[(2′-*O*-acetyl- *α*-l-arabinopyranosyl)oxy]-3-oxotaraxast-20-ene-11,28-diyl diacetate	NMR	*D. asper* (roots) [12]
2.	2′-*O*-acetyl-akebia saponin D; 2′-*O*-acetyl- 2′-*O*-acetyl-3-*O*-*α*-l-arabinopyranosyl-23-hydroxyolea- 12-en-28-oic acid; 3-*O*-(2-*O*-acetyl)-α-l-arabinopyranosyl-hederagenin 28-*O*-*β*-d-glucopyranosyl-(1-6)-*β*-d-glucopyranoside	HPLC-ESI-QTOF-MS/MS [93], R-ESI-MS, ^1^H NMR, ^13^C NMR [12,22,96]	*D. asper* (roots) [12,22,93,96]
3.	2*α*-hydroxyursolic acid	NMR	*D. asper* (roots) [12]
4.	2*α*-hydroxy- 3*β*-*O*-trans-feruloyloxy-olean-12-en-28-oic acid	NMR	*D. asper* (roots) [12]
5.	2*α*,3*β*-dihydroxy- 23-norolea-4(24),12-dien-28-oic acid	NMR	*D. asper* (roots) [12]
6.	2*α*,3*β*-dihydroxy-23-norurs-4(24),11,13(18)-trien-28-oic acid	HR-ESI-MS, ^1^H NMR, ^13^C NMR, ^1^H-^1^H COSY, ROESY	*D. asper* (roots) [12]
7.	2*α*,3*β*-dihydroxy- 24-norurs-4(23),12-dien-28-oic acid	NMR	*D. asper* (roots) [12]
8.	2*α*,3*β*,24-trihydroxy-23-norurs-12-en-28-oic acid	HR-ESI-MS, ^1^H NMR, ^13^C NMR, DEPT, ^1^H-^1^H COSY, HMBC, NOESY	*D. asper* (roots) [12]
9.	2*α*,23*α*-dihydroxy-3*β*-*O*-trans-feruloyloxy- olean-12-en-28-oic acid	NMR	*D. asper* (roots) [12]
10.	2′,3′-*O*-diacetyl-3-*O*-*α*-l-arabinopyranosyl-23-hydroxyolea-12-en-28-oic acid	HR-ESI-MS, ^1^H NMR, ^13^C NMR, HMBC	*D. asper* (roots) [12]
11.	2′,4′-*O*-diacetyl-3-*O*-*α*-l-arabinopyranosyl-23-hydroxyolea-12-en-28-oic acid	HR-ESI-MS, ^1^H NMR, ^13^C NMR, HMBC	*D. asper* (roots) [12]
12.	3*β*-*O*-trans-feruloyl-2*α*-hydroxy-urs-12-en-28-oic acid	NMR	*D. asper* (roots) [12]
13.	3*β*- *O*-trans-feruloyl-2*α*,23*α*-dihydroxy-urs-12-en-28-oic acid	NMR	*D. asper* (roots) [12]
14.	3*β*-hydroxy-24-norurs- 4(23),12-dien-28-oic acid	NMR	*D. asper* (roots) [12]
15.	3-*O*-α-l-arabinopyranosylhederagenin 28-*O*-*β*-d-glucopyranoside	NMR	*D. asper* (roots) [96]
16.	3-*O*-*α*-l-arabinopyranosyl-28-*O*-*β*-d-glucopyranosyl-(1→6)-*β*-d-glucopyranosyloleanolic acid	NMR [96], HPLC-ESI-QTOF-MS/MS [93]	*D. asper* (roots) [92,93,96]
17.	3-*O*-*β*-d-Glucopyranosyl-(1→3)-α-l-rhamnopyranosyl-(1→2)-α-l-arabinopyranosyl- 23-hydroxyolean-18-en-28-oic acid 28-*O-β*-Dglucopyranosyl-(1→6)-*β*-d-glucopyranosyl ester	HR-ESI-MS, 1D-TOCSY, 2D-HSQC, TOCSY-HSQC, COSY, HMBC	*D. asper* (roots) [96]
18.	3-*O*-[*β*-d-xylopyranosyl- (1→4)-*β*-d-glucopyranosyl-(1→4)][α-l-rhamnopyranosyl-(1→3)]-*β*- d-glucopyranosyl-(1→3)-α-l-rhamnopyranosyl-(1→2)-α-l-arabinopyranosylhederagenin	NMR	*D. asper* (roots) [96]
19.	3′-*O*-acetyl-akebia saponin D; 3′-*O*-acetyl-3-*O*-α-l-arabinopyranosylhederagenin 28-*O*-*β*-d-glucopyranosyl-(1→6)-*β*-d-glucopyranoside; 3′-*O*-acetyl-3- *O*-*α*-l-arabinopyranosyl-23-hydroxyolea-12-en-28-oic acid; 3-*O*-(3-*O*-acetyl)-α-l-arabinopyranosyl-hederagenin 28-*O*-*β-*d-glucopyranosyl-(1-6)-*β*-d-glucopyranoside	HR-ESI-MS, ^1^H NMR, ^13^C NMR [12,22,96], HPLC-ESI-QTOF-MS/MS [93]	*D. asper* (roots) [12,22,93,96]
20.	4′-*O*-acetyl-akebia saponin D (asperosaponin Ⅳ) ^a^; 4′-*O*-acetyl-3-*O*-α-l-arabinopyranosylhederagenin 28-*O*- *β*-d-glucopyranosyl-(1→6)-*β*-d-glucopyranoside; 4′-*O*-acetyl-3-*O*-*α*-l-arabinopyranosyl-23-hydroxyolea- 12-en-28-oic acid; 3-*O*-(4-*O*-acetyl)- α-l-arabinopyranosyl-hederagenin 28-*O*-*β*-d-glucopyranosyl-(1-6)-*β*-d-glucopyranoside	HPLC-ESI-QTOF-MS/MS [93], HR-ESI-MS, ^1^H NMR, ^13^C NMR [12,22,96]	*D. asper* (roots) [12,22,92,93,96]
21.	11*α*,12*α*-epoxy-3,6*β*-dihydroxy-24-norurs-3-en-2-on- (28→13)-olide	NMR	*D. asper* roots) [12]
22.	23*α*-hydroxy-olean-12-en-3- one	NMR	*D. asper* (roots) [12]
23.	25-acetoxy-28-dehydroxyrubiarbonone E	HR-ESI-MS, 1D NMR, 2D NMR, ^1^H-^1^H COSY, HMBC, NOESY	*D. asper* (roots) [12]
24.	Akebia saponin PA (cauloside A; leontoside A; 3-*O*-α-l-arabinopyranosyl hederagenin) ^a^	HPLC-ESI-QTOF-MS/MS [93], FAB-MS, 1D NMR, 2D NMR [21,22], HR-ESI-MS [22]	*D. asper/D. asperoides* (roots) [27,93] *D. asper* (roots) [6,21,22] *D. asper* (roots) [12]
25.	Akebia saponins X-Y	HPLC-ESI-QTOF-MS/MS [93]	*D. asper* (roots) [93]
26.	Asperosaponin B		*D. asper* (roots) [93]
27.	Asperosaponin E (3-*O-β*-d-glucopyranosyl-(1→4)-[a-l-rhamnopyranosyl-(1→6)]-*β*-d-glucopyranosyl-(1→3)-a-l-rhamnopyranosyl-(1→2)-a-l-arabinopyranosyl oleanolic acid)	HPLC-ESI-QTOF-MS/MS [93]	*D. asper* (roots) [93]
28.	Asperosaponin F (3-*O*-α-l-rhamnopyranosyl-(→›6)-*β*-d-glucopyranosyl-(1→3)-α-l-rhamnopyranosyl-(1→2)-α-l-arabinopyranosyl oleanolic acid)	HPLC-ESI-QTOF-MS/MS [93]	*D. asper* (roots) [93]
29.	Asperosaponin G (3-*O*-*β*-glucopyranosyl-(1→3)-α-l-rhamnopyranosyl-(1→2)-α-l-arabinopyranosyl oleanolic acid)	HPLC-ESI-QTOF-MS/MS [93]	*D. asper* (roots) [93]
30.	Asperosaponin V	UPLC-Q-TOF-MS	*D. asper* (roots) [66]
31.	Asperosaponin VI (akebia saponin D) ^a^ (3-*O*-α-l-arabinopyranosyl hederagenin-28-*β*-d-glucopyranoside-(1→6)- *β*-d-glucopyranoside)	UHPLC-MS/MS [65], UHPLC-Q-TOF-MS [66], LC-ESI-MS [90], HPLC-ESI-QTOF-MS/MS [93], HPLC-DAD [95]	*D. asper*/*D. asperoides* (roots) [65,66,89,90,92,93,95]
32.	Colchiside (3-*O-β-*d-xylopyranosyl-23-*O-β-*d-glucopyranosyl-28-*O-β-*d-(6-*O*-acetyl)- glucopyranosyl hederagenin)	1D NMR, 2D NMR, DEPT, TOCSY, HMQC, HMBC	*D. asper* (roots) [21]
33.	Dipsacoside A	UPLC-Q-TOF-MS	*D. asper* Wall. (roots) [66]
34.	Dipsacoside B	UHPLC-MS/MS [65]	*D. asperoides* (roots) [27,65]
35.	Dipsacus saponin A	HR-ESI-MS, ^1^H NMR, ^13^C NMR [22]	*D. asper* (roots) [6,22,92]
36.	Dipsacus saponin B	UPLC-Q-TOF-MS [66], HPLC-ESI-QTOF-MS/MS [93]	*D. asper*/*D. asperoides* (roots) [6,27,66,93]
37.	Dipsacus saponin C	ESI-QTOF-MS/MS [93]	*D. asper*/*D. asperoides* (roots) [6,27,91,93]
38.	Dipsacus saponins J-K	ESI-QTOF-MS/MS [93]	*D. asper* (roots) [6,93]
39.	Dipsacus saponin L (3-*O-*α-l-rhamnopyranosyl-(1→2)-α-l-arabinopyranosyl hederagenin)	ESI-QTOF-MS/MS	*D. asper* (roots) [93]
40.	Dipsacus saponin M (3-*O*-α-l-rhamnopyranosyl-(1→2)-α-l-arabinopyranosyl hed-eragenin 28-*O-β*-d-glucopyranosyl-(1→6)-*β*-d-glucopyranosyl)	ESI-QTOF-MS/MS	*D. asper* (roots) [93]
41.	Dipsacus saponin N (3-*O-β*-d-xylopyranosyl-(1→3)-α-l-rhamnopyranosyl-(1→2)-α-l-arabinopyranosyl hederagenin)	ESI-QTOF-MS/MS	*D. asper* (roots) [93]
42.	Dipsacus saponin O	ESI-QTOF-MS/MS	*D. asper* (roots) [93]
43.	Dipsacus saponin P (3-*O-β*-d-xylopyranosyl-(1→3)-α-l-rhamnopyranosyl-(1→2)-α-l-arabinopyranosyl hederagenin 28-*O-β*-d-glucopyranosyl-(1→6)-*β*-d-glucopyranosyl)	ESI-QTOF-MS/MS	*D. asper* (roots) [93]
44.	Dipsacus saponin R (3-*O*-α-l-rhamnopyranosyl(1→3)-*β*-d-glucopyranosyl (1→3)-α-l-rhamnopyranosyl-(1→2)-α-l-arabinopyranosyl-hederagenin)	ESI-QTOF-MS/MS	*D. asper* (roots) [93]
45.	Dipsacus saponin V	-	*D. asper* [6]
46.	Dipsacus saponin VI	HR-ESI-MS, ^1^H NMR, ^13^C NMR [22]	*D. asper* (roots) [6,22]
47.	Dipsacus saponin VII	-	*D. asper* [6]
48.	Dipsacus saponins IX-XI	-	*D. asper* [6]
49.	Dipsacus saponin XII	ESI-QTOF-MS/MS [93]	*D. asper* (roots) [6,93]
50.	Dipsacus saponin XIII	-	*D. asper* [6]
51.	Elmalienoside B	NMR	*D. asper* (roots) [96]
52.	α-hederin	-	*D. asper* [6]
53.	Hederagenin	-	*D. asper* [6]
54.	Hederagenin 28- *O*-*β*-d-glucopyranosyl-(1→6)-*β*-d-glucopyranoside	NMR	*D. asper* (roots) [96]
55.	Hederagonic acid	NMR	*D. asper* (roots) [12]
56.	HN saponin F	ESI-QTOF-MS/MS [93]	*D. asper* (roots) [6,93]
57.	Hookeroside A-B	NMR	*S. tschilliensis* (whole plants) [7,51]
58.	Japondipsaponin E1	-	*D. japonicus* (roots) [5,88]
59.	Kalopanax saponin A	-	*D. asper* (roots) [5]
60.	Oleanolic acid	^1^H NMR, ^13^C NMR [82], GC-MS [49]	*D. asper* (roots) [6,89] *S. arenaria* (aerial parts) [82] *S. stellata* (aerial parts) [49] *S. tschilliensis* (flowers) [94]
61.	Macranthoidin A	ESI-QTOF-MS/MS [93], NMR [12,96]	*D. asper* (roots) [6,12,93,96]
62.	Macranthoside B	-	*D. asper* [6]
63.	Maslinic acid	NMR	*D. asper* (roots) [12]
64.	Maslinic acid-pentosyl-rhamnosyl-glucoside	LC-MS/MS	*S. atropurpurea* subsp. *maritima* (leaves) [81]
65.	Oleanolic acid-pentosyl-rhamnosyl-glucosyl-glucosyl-di-glucoside	LC-MS/MS	*S. atropurpurea* subsp. *maritima* (leaves) [81]
66.	Oleanolic acid-pentosyl-rhamnosyl-pentosyl-glucosyl-di-glucoside	LC-MS/MS	*S. atropurpurea* subsp. *maritima* (leaves) [81]
67.	Palustroside III (3-*O*-[*β*-d-glucopyranosyl-(1→2)-*β*-d-glucuronopyranosyl]-28-*O*-[*β*-d-glucopyranosyl]-hederagenin)	-	*S. stellata* (whole plants) [7,48]
68.	Saponin XII (3-*O*-[*β*-d-glucopyranosyl(1→4)][α-l-rhamnopyranosyl(1→3)]- emph-*β-*d-glucopyranosyl(1→3)-α-l-rhamnopyranosyl(1→2)-α-arabinopyranosyl hederagenin 28-*O*-*β*-d-glucopyranosyl(1→6)-*β*-d-glucopyranoside)	1D NMR, 2D NMR, DEPT, HR-ESI-MS	*D. japonicus* (roots) [40]
69.	Scabiosaponin A (3-*O*-*β*-d-glucopyranosyl-(1→4)-*β*-d-xylopyranosyl-(1→3)-α-l-rhamnopyranosyl-(1→2)-α-l-arabinopyranosyloleanolic acid 28-*O*-*β*-d-glucopyranosyl-(1→6)-*β*-d-glucopyranoside)	2D NMR, DEPT, DQF-COSY, TOCSY, HMQC, HMQC-TOCSY, HMBC, NOESY [51]	*S. tschilliensis* (whole plants) [7,51]
70.	Scabiosaponin B (3-*O*-*β*-d-xylopyranosyl-(1→4)-*β*-d-glucopyranosyl-(1→4)-*β*-d-xylopyranosyl-(1→3)-α-l-rhamnopyranosyl-(1→2)-α-l-arabinopyranosyloleanolic acid 28-*O*-*β*-d-glucopyranosyl-(1→6)-*β*-d-glucopyranoside)	2D NMR, DEPT, DQF-COSY, TOCSY, HMQC, HMQC-TOCSY, HMBC, NOESY [51]	*S. tschilliensis* (whole plants) [7,51]
71.	Scabiosaponin C (3-*O*-[*β*-d-glucopyranosyl-(1→4)-*β*-d-xylopyranosyl-(1→3)-α-l-rhamnopyranosy-(1→2)][*β*-d-glucopyranosyl-(1→4)]-α-l-arabinopyranosyloleanolic acid 28-*O*-*β*-d-glucopyranosyl-(1→6)-*β*-d-glucopyranoside)	2D NMR, DEPT, DQF-COSY, TOCSY, HMQC, HMQC-TOCSY, HMBC, NOESY [51]	*S. tschilliensis* (whole plants) [7,51]
72.	Scabiosaponin D (3-*O*-[α-l-rhamnopyranosyl-(1→2)][*β*-d-glucopyranosyl-(1→4)]-α-l-arabinopyranosyloleanolic acid 28-*O*-*β*-d-glucopyranosyl-(1→6)-*β*-d-glucopyranoside)	2D NMR, DEPT, DQF-COSY, TOCSY, HMQC, HMQC-TOCSY, HMBC, NOESY [51]	*S. tschilliensis* (whole plants) [7,51]
73.	Scabiosaponin E (3-*O*-*β*-d-xylopyranosyl-(1→3)-α-l-rhamnopyranosyl-(1→2)-*β*-d-xylopyranosyloleanolic acid 28-*O*-*β*-d-glucopyranosyl-(1→6)- *β*-d-glucopyranoside)	2D NMR, DEPT, DQF-COSY, TOCSY, HMQC, HMQC-TOCSY, HMBC, NOESY [51]	*S. tschilliensis* (whole plants) [7,51]
74.	Scabiosaponin F (3-*O*-*β*-d-glucopyranosyl- (1→3)-α-l-rhamnopyranosyl-(1→2)-*β*-d-xylopyranosyloleanolic acid 28-*O*-*β*-d-glucopyranosyl-(1→6)-β-d-glucopyranoside)	2D NMR, DEPT, DQF-COSY, TOCSY, HMQC, HMQC-TOCSY, HMBC, NOESY [51]	*S. tschilliensis* (whole plants) [7,51]
75.	Scabiosaponin G (3-*O*-*β*-d-glucopyranosyl-(1→4)-*β*-d-glucopyranosyl-(1→3)-α-l-rhamnopyranosyl-(1→2)-*β*-d-xylopyranosyloleanolic acid 28-*O*-*β*-d-glucopyranosyl-(1→6)-*β*-d-glucopyranoside)	2D NMR, DEPT, DQF-COSY, TOCSY, HMQC, HMQC-TOCSY, HMBC, NOESY [51]	*S. tschilliensis* (whole plants) [7,51]
76.	Scabiosaponin H (3-*O*-*β*-d-glucopyranosyl-(1→4)-*β*-d-glucopyranosyl-(1→3)-α-l-rhamnopyranosyl-(1→2)-α-L-arabinopyranosylpomolic acid 28-*O*-*β*-d-glucopyranosyl-(1→6)-*β*-d-glucopyranoside)	2D NMR, DEPT, DQF-COSY, TOCSY, HMQC, HMQC-TOCSY, HMBC, NOESY [51]	*S. tschilliensis* (whole plants) [7,51]
77.	Scabiosaponin I (3-*O*-*β*-d-glucopyranosyl-(1→3)-α-l-rhamnopyranosyl-(1→2)-α-l-arabinopyranosylpomolic acid 28-*O*-*β*-d-glucopyranosyl-(1→6)-*β*-d-glucopyranoside)	2D NMR, DEPT, DQF-COSY, TOCSY, HMQC, HMQC-TOCSY, HMBC, NOESY [51]	*S. tschilliensis* (whole plants) [7,51]
78.	Scabiosaponin J (3-*O*-*β*-d-glucopyranosyl-(1→3)-α-l-rhamnopyranosyl-(1→2)-α-l-arabinopyranosylsiaresinolic acid 28-*O*-*β*-d-glucopyranosyl-(1→6)-*β*-d-glucopyranoside)	2D NMR, DEPT, DQF-COSY, TOCSY, HMQC, HMQC-TOCSY, HMBC, NOESY [51]	*S. tschilliensis* (whole plants) [7,51]
79.	Scabiosaponin K (3-*O*-*β*-d-glucopyranosyl-(1→4)-*β*-d-xylopyranosyl-(1→3)-α-l-rhamnopyranosyl-(1→2)-α-l-arabinopyranosylsiaresinolic acid 28-*O*-*β*-d-glucopyranosyl-(1→6)-*β*-d-glucopyranoside)	2D NMR, DEPT, DQF-COSY, TOCSY, HMQC, HMQC-TOCSY, HMBC, NOESY [51]	*S. tschilliensis* (whole plants) [7,51]
80.	Scabiostellatoside A (3-*O*-[*β*-d-glucopyranosyl-(1→4)-*β-*d-xylopyranosyl-(1→3)-α-l-rhamnopyranosyl-(1→2)-α-l-arabinopyranonosyl]-28-*O*-[*β*-d-glucopyranosyl-(1→6)-*β*-d-glucopyranosyl]-oleanolic acid)	HR-ESI-MS, 1D NMR, 2D NMR, HMBC, COSY, TOCSY, HSQC, ROESY [48]	*S. stellata* (whole plants) [7,48]
81.	Scabiostellatoside B (3-*O*-α-l-rhamnopyranosyl-(1→3)-*β*-d-xylopyranosyl-(1→3)-α-l-rhamnopyranosyl-(1→2)-*β*-d-xylopyranosyl]-28-*O*-[*β*-d-glucopyranosyl-(1→6)-*β*-d-glucopyranosyl]-oleanolic acid)	HR-ESI-MS, 1D NMR, 2D NMR, HMBC, COSY, TOCSY, HSQC, HSQC-TOCSY, ROESY [48]	*S. stellata* (whole plants) [7,48]
82.	Scabiostellatoside C (3-*O*-[*β*-d-glucopyranosyl-(1→4)-α-l-rhamnopyranosyl-(1→3)-*β*-d-xylopyranosyl-(1→3)-α-l-rhamnopyranosyl-(1→2)-a-l-arabinopyranosyl]-28-*O*-[*β*-d-glucopyranosyl-(1→6)-*β*-d-glucopyranosyl]-oleanolic acid)	HR-ESI-MS, 1D NMR, 2D NMR, HMBC, COSY, TOCSY, HSQC, HSQC-TOCSY, ROESY [48]	*S. stellata* (whole plants) [7,48]
83.	Scabiostellatoside D (3-*O*-[*β*-d-glucopyranosyl-(1→4)-α-l-rhamnopyranosyl-(1→3)-*β*-d-xylopyranosyl-(1→3)-α-l-rhamnopyranosyl-(1→2)-*β*-d-xylopyranonosyl]-28-*O*-[*β*-d-glucopyranosyl-(1→6)-*β*-d-glucopyranosyl]-oleanolic acid)	HR-ESI-MS, 1D NMR, 2D NMR, HMBC, COSY, TOCSY, HSQC, HSQC-TOCSY, ROESY [48]	*S. stellata* (whole plants) [7,48]
84.	Scabiostellatoside E (3-*O*-[*α*-l-rhamnopyranosyl- (1→3)-*β*-d-xylopyranosyl-(1→3)-α-l-rhamnopyranosyl-(1→2)-{*β*-d-glucopyranosyl-(1→4)-}α-l-arabinopyranosyl]-28-*O*-[*β*-Dglucopyranosyl-(1→6)-*β*-d-glucopyranosyl]-oleanolic acid)	HR-ESI-MS, 1D NMR, 2D NMR, HMBC, COSY, TOCSY, HSQC, HSQC-TOCSY, ROESY [48]	*S. stellata* (whole plants) [7,48]
85.	Scabiostellatoside F (3-*O*-[*β*-d-glucopyranosyl-(1→4)-*β*-d-glucopyranosyl-(1→4)-β-d-glucopyranosyl-(1→4)-α-l-rhamnopyranosyl-(1→3)-*β*-d-xylopyranosyl-(1→3)-α-l-rhamnopyranosyl-(1→2)-*β*-d-xylopyranosyl]- oleanolic acid)	HR-ESI-MS, 1D NMR, 2D NMR, HMBC, COSY, TOCSY, HSQC, HSQC-TOCSY, ROESY [48]	*S. stellata* (whole plants) [7,48]
86.	Scabiostellatoside G (3-*O*-[*β*-d-glucopyranosyl-(1→2)-*β*-d-glucuronopyranosyl]-28-*O*-[*β*-d-glucopyranosyl-(1→6)-*β*-d-glucopyranosyl]-hederagenin)	HR-ESI-MS, 1D NMR, 2D NMR, HMBC, COSY, TOCSY, HSQC, HSQC-TOCSY, ROESY [48]	*S. stellata* (whole plants) [7,48]
87.	Scabiostellatoside H (3-*O*-[α-l-rhamnopyranosyl-(1→2)-*β*-d-glucuronopyranosyl]-28-*O*-[*β*-d-glucopyranosyl]-asiatic acid)	HR-ESI-MS, 1D NMR, 2D NMR, HMBC, COSY, TOCSY, HSQC, HSQC-TOCSY, ROESY [48]	*S. stellata* (whole plants) [7,48]
88.	Urceolide	-	*S. tschilliensis* (flowers) [94]
89.	Ursolic acid	NMR [12], GC-MS [49]	*D. asper* (roots) [12] *S. stellata* (whole plants) [7,48,49]

^a^ synonymous names of compounds are given in accordance with PubChem database [109].

#### *Iridoids* 

Iridoids were found in both genera (Table 3). The first iridoids isolated from *D. asper* were loganin and sweroside [6]. The presence of iridoid glycosides including loganin and loganic acid was widespread in the roots or leaves of *Dipsacus* spp. [6,22,27,33,34,65,66,89,92,93,95,96,97,99] and flowers of *S. atropurpurea* subsp. *maritima* [7,84] (Table 3). Dipsanosides A-N, dipsasperoside A and B, lisianthioside, triplostoside A, and cocculoside were characteristic only for roots of *D. asper* [6,13,22,89,92,93,96,98,99,100]. Sweroside and sylvestrosides III-IV were found in two *Dipsacus* species: *D. asper* roots and *D. fullonum* leaves and roots [6,22,27,33,34,65,66,92,93,96,99]. Sweroside and sylvestrosides I and II were also detected in some *Scabiosa* species, such as *S. stellata* whole plants, *S. tschilliensis*, and *S. atropurpurea* subsp. *maritima* flowers (Table 3) [7,75,84,94]. 7-*O*-caffeoyl-sylvestroside I and 7-*O*-(*p*-coumaroyl)-sylvestroside I were isolated as new compounds from the whole plants of *S. stellata* [7,75]. Interestingly, three iridoid-like compounds, viz., eustomoside, eustomoruside, and septemfidoside, were identified only in *S. stellata* [7,75]. It should be also noted that these iridoids have not been previously described in Caprifoliaceae [75]. Atropurpurin A-B and secologanin-methyl-hemiacetal were reported for the first time in *Scabiosa* spp. in *S. atropurpurea* subsp. *maritima* [78]. Lehbili et al. [75] suggested that *Dipsacus* and *Scabiosa* are closely related to one another; septemfidoside, sylvestroside I, and its derivatives (e.g., 7-*O*-caffeoyl-sylvestroside I and 7-*O*-[*p*-coumaroyl]-sylvestroside I) isolated from *S. stellata* whole plants are closely related to the *bis*-iridoids, cantleyoside identified in *S. atropurpurea* subsp. *maritima* [7,84], *Dipsaci radix* [6,89,92,93,97,99], and *D. fullonum* [33] as well as dipsanosides C-G detected in *D. asper* [6,89]. The *bis*-iridoids identified in *Dipsacus* spp. possess a secoiridoid/iridoid subtype skeleton consisting of secologanic acid condensed to the 7-OH of loganin or loganin-like iridoids [89].

The levels of the main iridoids in *Dipsaci radix*, e.g., loganic acid, loganin, sweroside, and dipsanosides A and B, varied according to location and collection time. The highest level of loganic acid was 31.223 mg/g; loganin, 4.411 mg/g; sweroside, 8.364 mg/g; dipsanoside A, 4.513 mg/g; and dipsanoside B, 7.426 mg/g [99]. Du et al. [95] also demonstrated that the content of loganic acid (0.71–1.10%) and loganin (0.29–0.61%) varied between samples from different origins in China. In addition, the level of some iridoids in the leaves and roots including loganic acid, loganin, sweroside, sylvestroside III, and cantleyoside detected in *D. fullonum* varied according to the time of year of harvesting [33]. The leaves of the plants collected in the first year of vegetation contained a higher total iridoid content than the roots and leaves harvested in the second year. The most abundant iridoids were sylvestroside III in the leaves and cantleyoside in the roots. Moreover, sweroside (0.67 mg/g dry weight) and sylvestroside III (34.8 mg/g d.w.) were present in higher amounts in leaves, while the levels of loganic acid (5.27 mg/g d.w.), loganin (3.02 mg/g d.w.), and cantleyoside (21.41 mg/g d.w.) were higher in the roots [33].

**Table 3:** Iridoids identified in *Dipsacus* and *Scabiosa* species.

No.	Compound Name	Species (Part of Plant)
1.	3′-*O*-*β-*d-glucopyranosyl sweroside	*D. asper* (roots) [89]
2.	6′-*O*-*β*-d-apiofuranosyl sweroside	*D. asper* (roots) [89,99]
3.	7-*O*-caffeoyl-sylvestroside I	*S. stellata* (whole plants) [7,75]
4.	7-*O*-(*p*-coumaroyl)-sylvestroside I	*S. stellata* (whole plants) [7,75]
5.	Atropurpurin A	*S. atropurpurea* subsp. *maritima* (whole plants) [78]
6.	Atropurpurin B	*S. atropurpurea* subsp. *maritima* (whole plants) [78]
7.	Cantleyoside	*D. asper* (roots) [6,89,92,93,97,99] *D. fullonum* (leaves and roots) [33] *S. atropurpurea* subsp. *maritima* (roots) [7,84]
8.	Cocculoside	*D. asper* (roots) [96]
9.	Dipsanoside A and dipsanoside B	*D. asper* (roots) [6,22,92,93,99]
10.	Dipsanosides C-G	*D. asper* (roots) [6,89]
11.	Dipsanoside H	*D. asper* [6]
12.	Dipsanosides M-N	*D. asper* (roots) [6,13]
13.	Dipasperoside A	*D. asper* (roots) [100]
14.	Dipasperoside B	*D. asper* (roots) [98]
15.	Eustomoruside	*S. stellata* (whole plants) [7,75]
16.	Eustomoside	*S. stellata* (whole plants) [7,75]
17.	Rhamnopyranosyl-cantleyoside	*D. asper* (roots) [99]
18.	Lisianthioside	*D. asper* (roots) [6,89]
19.	Loganic acid	*D. asper* (roots) [6,22,65,66,92,93,95,99] *D. fullonum* (leaves, roots) [33,34] *S. atropurpurea* subsp. *maritima* (flowers) [7,84]
20.	Loganic acid ethyl ester	*D. asper* (roots) [97]
21.	Loganin	*D. asper/D. asperoides* (roots) [6,22,27,65,66,89,92,93,95,96,97,99] *D. fullonum* (leaves, roots) [33,34] *S. atropurpurea* subsp. *maritima* (flowers) [7,84]
22.	Secologanin-methyl-hemiacetal	*S. atropurpurea* subsp. *maritima* (whole plants) [78]
23.	Septemfidoside	*S. stellata* (whole plants) [7,75]
24.	Sweroside	*D. asper*/*D. asperoides* (roots) [6,22,27,65,66,92,93,96,99] *D. fullonum* leaves, roots) [33] *S. stellata* (whole plants) [7,75] *S. atropurpurea* subsp. *maritima* (flowers) [7,84] *S. tschilliensis* (flowers) [94]
25.	Sylvestroside I	*D. asper* (roots) [22,92,99] *S. stellata* (whole plants) [7,75] *S. tschilliensis* (flowers) [94]
26.	Sylvestroside II	*S. tschilliensis* (flowers) [94]
27.	Sylvestroside III	*D. asper* [6] *D. fullonum* (leaves, roots) [33,34]
28.	Sylvestroside IV	*D. asper* (roots) [6] *D. fullonum* (leaves) [34]
29.	Triplostoside A	*D. asper* (roots) [6,89,92,93,99]

### 3.2. Phenolic Acids

Many phenolic acids have been noted in *Dipsacus* and *Scabiosa* species (Table 4) [6,24,27,29,33,34,43,46,53,65,66,73,74,75,76,81,89,92,93,94,95,97,99,101]. Mainly derivatives of hydroxycinnamic acid were identified, with chlorogenic acid as the most abundant component. This phenolic acid was detected in *Dipsaci radix* [6,27,29,65,93,95,97,99], *D. fullonum* leaves and roots [33,34,101], *S. comosa* and *S. tschiliensis* inflorescences [53,74], *S. atropurpurea* stems [46], and *S. atropurpurea* subsp. *maritima* leaves [81]. In addition, other mono-caffeoylquinic acid derivatives (1-*O*-caffeoylquinic acid, 3-*O*-caffeoylquinic acid methyl ester, and 4-*O*-caffeoylquinic acid) and dicaffeoylquinic acid derivatives (3,4-di-*O*-caffeoylquinic acid, 3,5-di-*O*-caffeoylquinic acid, 4,5-di-*O*-caffeoylquinic acid, and their methyl derivative) have been identified (Table 4) [6,24,27,33,43,65,66,74,75,76,81,92,93,95,99]. The hydroxycinnamic acid derivatives included caffeic acid, caffeic acid methyl ester, *p*-coumaric acid, *p*-coumaric acid 3-glucoside, 2,6-dihydroxycinnamic acid, 5-*O*-ferruloylqunic acid, *p*-hydroxycinnamic acid, and sinapic acid [6,29,46,53,73,74,76,81,89,92,93,94,95,97,99]. The benzoic acid derivatives were 3,4-dihydroxybenzoic acid, protocatechuic acid, protocatechuic acid 3-glucoside, and vanillic acid [6,74,81,89,92] (Table 4).

The phenolic acid content in *Dipsaci radix* varied according to the geographical region of China [95,99]. The total level of phenolic acids ranged from 0.98 mg/g to 49.55 mg/g, with the highest level observed in plant material collected from Guizhou Province [99]. The predominant phenolic acid was chlorogenic acid (0.186–19.174 mg/g; 2.02–8.28%) [95,99], which was also an abundant phenolic in *D. fullonum* [33]. However, qualitative differences were reported between the leaves and the roots, with the highest level being found in the leaves obtained from plants in the second year of vegetation (28.44 mg/g d.w.). Wang et al. [53] also found differences in the content of chlorogenic acid in plants of *S. tschiliensis* between the pre-flowering, flowering, and fruiting stages. The level was also dependent on the extraction solvent; the richest source of chlorogenic acid, 45.35 mg/g d.w., was found in the ethyl acetate fraction from plants at the pre-flowering stage.

**Table 4:** Phenolic acids identified in *Dipsacus* and *Scabiosa* species.

No.	Compound Name	Species (Part of Plant)
1.	1-*O*-caffeoylquinic acid	*D. asper* (roots) [6] *S. stellata* (whole plants) [76]
2.	2′-*O*-caffeoyl-d-glucopyranoside ester	*D. asper* (roots) [89]
3.	2,6-dihydroxycinnamic acid	*D. asper* (roots) [6,89]
4.	3-*O*-caffeoylquinic acid methyl ester	*S. atropurpurea* subsp. *maritima* (leaves) [81] *S. atropurpurea* L. (aerial parts) [43]
5.	3,4-di-*O*-caffeoylquinic acid (isochlorogenic acid B) ^a^	*D. asper* (roots) [6,24,66,92,93,95,99] *S. atropurpurea* sbsp. *maritima* (leaves) [81] *S. comosa* (inflorescences) [74] *S. stellata* (whole plants) [76] *S. tschilliensis* (inflorescences) [74]
6.	3,4-dihydroxybenzoic acid	*D. asper* (roots) [92]
7.	3,5-di-*O*-caffeoylquinic acid (isochlorogenic A) ^a^	*D. asper*/*D. asperoides* (roots) [6,24,27,65,66,92,93,95,99] *D. fullonum* (leaves, roots) [33] *S. atropurpurea* subsp. *maritima* (leaves) [6] *S. comosa* (inflorescences) [74] *S. stellata* (whole plants) [75,76] *S. tschilliensis* (inflorescences) [74]
8.	4*-O*-caffeoylquinic acid (cryptochlorogenic acid) ^a^	*D. asper* (roots) [6,65,66] *D. fullonum* (leaves, roots) [33] *S. atropurpurea* subsp. *maritima* (leaves) [81] *S. stellata* (whole plants) [76]
9.	4,5-di-*O*-caffeoylquinic acid (isochlorogenic acid C) ^a^	*D. asper* (roots) [6,24,92,93,95,99] *S. comosa* (inflorescences) [74] *S. stellata* (whole plants) [75,76] *S. tschilliensis* (inflorescences) [74]
10.	5-*O*-feruloylquinic acid	*S. stellata* (whole plants) [76]
11.	5-*O*-*p*-coumaroylquinic acid	*S. stellata* (whole plants) [76]
12.	Caffeic acid	*D. asper*/*D. asperoides* (roots) [6,29,89,92,93,95,97,99] *S. comosa* (inflorescences) [74] *S. tschilliensis* (inflorescences) [53,74] *S. atropurpurea* (stems) [46]
13.	Caffeic acid methyl ester	*S. tschilliensis* (flowers) [94]
14.	Chlorogenic acid	*D. asper/D. asperoides* (roots) [6,27,29,65,93,95,97,99] *D. fullonum* (leaves, roots) [33,34,101] *S. atropurpurea* subsp. m*aritima* (leaves) [81] *S. atropurpurea* (stems) [46] *S. comosa* (inflorescences) [74] *S. tschilliensis* (inflorescences) [53,74]
15.	Methyl 3,4-di-*O*-caffeoylquinate	*D. asper* (roots) [6,24]
16.	Methyl 3,5-di-*O*-caffeoylquinate	*D. asper* (roots) [6,24]
17.	Methyl 4,5-di-*O*-caffeoylquinate	*D. asper* (roots) [6,24]
18.	Neochlorogenic acid	*D. fullonum* (leaves, roots) [33] *S. atropurpurea* subsp. *maritima* (leaves) [81]
19.	*p*-coumaric acid	*S. comosa* (inflorescences) [74] *S. tschilliensis* (inflorescences) [74] *S. atropurpurea* (stems) [46] *S. arenaria* (roots) [73]
20.	*p*-coumaric acid 3-glucoside	*S. atropurpurea*. subsp. *maritima* (leaves) [81]
21.	*p*-coumaroylquinic acid	*S. atropurpurea* subsp. *maritima* (leaves) [81]
22.	*p*-hydroxycinnamic acid	*S. atropurpurea* (stems) [46]
23.	Protocatechuic acid	*S. comosa* (inflorescences) [74] *S. tschilliensis* (inflorescences) [74]
24.	Protocatechuic acid 3-glucoside	*S. atropurpurea* subsp. *maritima* (leaves) [81]
25.	Quinic acid	*S. comosa* inflorescences) [74] *S. tschilliensis* inflorescences [74] *S. atropurpurea* subsp. *maritima* (leaves) [81]
26.	Sinapic acid	*S. stellata* (whole plants) [76]
27.	Vanillic acid	*D. asper* (roots) [6,89,92]

^a^ synonymous names of compounds are given in accordance with PubChem database [109].

### 3.3. Flavonoids

The *Scabiosa* genus is a rich source of flavonoids, which are identified mainly in the flowers but also in the leaves, stems, and roots (Table 5) [7,43,46,48,53,73,74,75,76,81,82,94]. Many studies note that apigenin, luteolin, and their derivatives are particularly common [7,43,46,74,75,76,81,82,94]. The new flavonoids identified for the first time in *Scabiosa* spp. were diosmetin-7-*O*-glucoside, luteolin-7,3′-diglucoside, luteolin 3′-glucoside, and quercetin 3,4′-diglucoside in *S. atropurpurea* subsp. *maritima* leaves [81]; quercimeritrin in *S. atropurpurea* stems; quercitrin, rutin, kaempferol-3-*O*-*β*-d-6-*O*-(*p*-hydroxycinnamoyl)-glucopyranoside, and kaempferol-3-*O*-*β*-d-(3,6-di-*p*-(hydroxycinnamoyl)-glucopyranoside in *S. tschilliensis* flowers [46,94]; and tamarixetin derivative 3-*β*-l-rhamnosyl-(1→2)[*β*-l-rhamnosyl-(1→6)]*β*-d-glucoside] and tiliroside in *S. stellata* whole plants [76]. Isoorientin, isovitexin, orientin, saponarin, and saponaretin were detected for the first time in *Dipsacus* spp., in *D. fullonum* leaves [33,34].

**Table 5:** Flavonoids identified in *Dipsacus* and *Scabiosa* species.

No.	Compound Name	Species (Part of Plant)
1.	3-*O*-[3-*O*-acetyl-6-*O*-*(p*-coumaroyl)-*β*-d-glucopyranosyl]-kaempferol	*S. stellata* (whole plants) [48]
2.	Apigenin	*S. tschilliensis* (flowers) [94] *S. comosa* (inflorescences) [74] *S. tschilliensis* (inflorescences) [74]
3.	Apigenin-2″-*O*-pentosyl-8-C-glucoside	*S. stellata* (whole plants) [76]
4.	Apigenin-4′-glucoside (apigenin-4′-*O*-β-d-glucopyranoside) ^a^	*S. comosa* (inflorescences) [74] *S. tschilliensis* (inflorescences/flowers) [74,94]
5.	Apigenin-7-arabino(1~6)-glucoside	*S. comosa* (inflorescences) [74] *S. tschilliensis* (inflorescences) [74]
6.	Apigenin-7-glucoside (apigenin-7*-O*-*β*-d-glucopyranoside) ^a^	*S. atropurpurea* subsp. *maritima* (leaves) [81] *S. comosa* (inflorescences) [74] *S. tschilliensis* (inflorescences/flowers) [74,94]
7.	Apigenin-7-*O*-rutinoside	*S. tschilliensis* (flowers) [94]
8.	Diosmetin-6(or 8)-*C*-glucoside	*S. stellata* (whole plants) [76]
9.	Diosmetin-7-*O*-glucoside	*S. atropurpurea* subsp. *maritima* (leaves) [81]
10.	Hyperin (hyperoside; quercetin 3-*O*-galactoside) ^a^	*S. atropurpurea* (stems) [46] *S. stellata* (whole plants) [7,75,76]
11.	Icariin	*S. tschiliensis* (inflorescences) [53]
12.	Isoorientin (luteolin-6-C-glucoside) ^a^	*D. fullonum* (leaves) [34] *D. sativus* L. (leaves) [42] *S. comosa* (inflorescences) [74] *S. stellata* (whole plants) [7,75,76] *S. tschilliensis* (inflorescences) [74]
13.	Isoquercitrin (quercetin 3-glucoside) ^a^	*S. atropurpurea* (stems) [46] *S. comosa* (inflorescences) [74] *S. tschilliensis* (inflorescences) [74]
14.	Isovitexin (saponaretin; apigenin-6-C-glucoside) ^a^	*D. fullonum* (leaves) [33,34] *D. sativus* (leaves) [42]
15.	Kaempferol-3-*O*-β-d-(3,6-di-*p*-(hydroxycinnamoyl)-glucopyranoside	*S. tschilliensis* (flowers) [94]
16.	Kaempferol-3-*O*-β-d-6-*O*-(*p*-hydroxycinnamoyl)-glucopyranoside	*S. tschilliensis* (flowers) [94]
17.	Kaempferol-3-*O*-rutinoside derivative	*S. stellata* (whole plants) [76]
18.	Lucenin-2 (luteolin-6,8-di-C-glucoside) ^a^	*S. stellata* (whole plants) [76]
19.	Luteolin	*S. atropurpurea* subsp. *maritima* (leaves) [81] *S. atropurpurea* (aerial parts, stems) [7,43,46] *S. comosa* (inflorescences) [74] *S. tschilliensis* (inflorescences/flowers) [74,94]
20.	Luteolin-2″-*O*-pentosyl-6-C-hexoside	*S. stellata* (whole plants) [76]
21.	Luteolin-4′-*O*-β-d-glucopyranoside (luteolin-4′-glucoside) ^a^	*S. comosa* (inflorescences) [74] *S. tschilliensis* (flowers) [74,94]
22.	Luteolin 3′-glucoside	*S. atropurpurea* subsp. *maritima* (leaves) [81]
23.	Luteolin hexoside	*S. atropurpurea* (stems) [46]
24.	Luteolin-6-C-glucoside-7-*O*-glucoside	*S. stellata* (whole plants) [76]
25.	Luteolin 7-rutinoside (luteolin-7-*O*-*β*-d-rutinoside) ^a^	*S. atropurpurea* (aerial parts) [43] *S. atropurpurea* subsp. *maritima* (leaves) [81]
26.	Luteolin-7,3′-diglucoside	*S. atropurpurea* subsp. *maritima* (leaves) [81]
27.	Luteolin 7-*O*-*β*-d-glucoside (cynaroside; luteolin-7-*O*-β-d-glucopyranoside) ^a^	*S. arenaria* (aerial parts) [82] *S. atropurpurea* (stems) [46] *S. atropurpurea* subsp. *maritima* (leaves/aerial parts) [43,81] *S. tschilliensis* (flowers) [81]
28.	Myricetin	*S. arenaria* (roots) [73]
29.	Orientin (luteolin 8-C-β-d-glucopyranoside, luteolin 8-C-glucoside) ^a^	*D. fullonum* (leaves) [33]
30.	Quercetin-3-rutinoside	*S. comosa* (inflorescences) [74] *S. tschilliensis* (inflorescences) [74]
31.	Quercetin 3,4′-diglucoside	*S. atropurpurea* subsp. *maritima* (leaves) [81]
32.	Quercimeritrin (quercetin 7-glucoside) ^a^	*S. atropurpurea* (stems) [46]
33.	Quercitrin (quercitin-3-*O*-rhamnoside) ^a^	*S. tschiliensis* (inflorescences) [53]
34.	Rutin (quercetin 3-rutinoside) ^a^	*S. tschiliensis* (inflorescences) [53]
35.	Saponarin (apigenin-6-C-glucoside-7-*O*-glucoside) ^a^	*D. fullonum* (leaves, roots) [33,34] *D. sativus* (leaves) [42]
36.	Swertiajaponin (isoorientin 7-methyl ether) ^a^	*S. stellata* (whole plants) [7,75]
37.	Tamarixetin derivative (3-*β*-l-rhamnosyl-(1→2)[*β*-l-rhamnosyl-(1→6)]β-d-glucoside	*S. stellata* (whole plants) [7,76]
38.	Tiliroside	*S. stellata* (whole plants) [7,76]

^a^ synonymous names of compounds are given in accordance with PubChem database [109].

### 3.4. Lignans

Lignans were identified only in *Dipsaci radix* and include prinsepiol, fraxiresinol, and their derivatives such as dipsalignans A-D [6,13,99]. *Dipsaci radix* also demonstrated derivatives of pinoresinol and syringaresinol (Table 6) [13,46,96,97,99]. Syringaresinol hexoside was detected as a new compound in *S. atropurpurea* stems [46].

**Table 6:** Lignans identified in *Dipsacus* and *Scabiosa* species.

No.	Compound Name	Species (Part of Plant)
1.	(7R, 8S, 7′R, 8′S)-fraxiresinol-4′-*O*-*β*-d-glucopyranoside	*D. asper* (roots) [99]
2.	(7R, 8S, 7′R, 8′S)-prinsepiol- 4-*O*-*β*-d-glucopyranoside	*D. asper* (roots) [99]
3.	(7R, 8S, 7′R, 8′S)-8-hydroxypinoresinol- 4′-*O*-*β*-d-glucopyranoside 8′-hydroxypinoresinol-4′-*O-β-*d-glucopyranoside	*D. asper* (roots) [96,99]
4.	(+)-1-hydroxy-2,6-bis-*epi*-pinoresinol	*D. asper* (roots) [6]
5.	(+)-8-hydroxy-7,7′-bis-*epi*-pinoresinol	*D. asper* (roots) [13]
6.	Dipsalignan A ((+)-8-hydroxy-7,7′-bis-*epi*-fraxiresinol)	*D. asper* (roots) [6,13]
7.	Dipsalignan B ((+)-(7*S*, 8*S*, 7′*R*, 8′*S*)-prinsepiol)	*D. asper* (roots) [6,13]
8.	Dipsalignan C ((+)-(7*S*, 8*S*, 7′*R*, 8′*S*)-5-methoxyprinsepiol)	*D. asper* (roots) [6,13]
9.	Dipsalignan D ((+)-(7*S*, 8*R*, 7′*S*, 8′*R*)-5-methoxyprinsepiol)	*D. asper* (roots) [6,13]
10.	Syringaresinol-4′,4″-*O*-bis-β-d-glucoside	*D. asper* (roots) [97]
11.	Syringaresinol hexoside	*S. atropurpurea* (stems) [46]

### 3.5. Polysaccharides

*Dipsacus* spp. also include polysaccharides [11,63,110,111]. The water-soluble polysaccharide from *D. asperoides* roots (ADAPW) has a molecular weight of 16 kDa and contains glucose, rhamnose, arabinose, and mannose in a molar ratio of 8.54:1.83:1.04:0.42 [11]. The polysaccharide WDRAP-1, with a molecular weight of 61 kDa, was composed of glucose, mannose, galactose, arabinose, and rhamnose in a molar ratio of 3.1:0.9:5.2:1.1:0.3. The predominant monosaccharides were glucose (29.2%) and galactose (49.1%) [63]. Xu et al. [110] found two polysaccharides in *D. asperoides* roots, DAI-1 and DAI-2, which consisted only of glucose and had respective molecular masses of 17 and 4 kDa. Sun et al. [111] isolated from *D. asper* roots a homogenous polysaccharide (DAP) with a molecular weight of 26.1 kDa that was composed of galactose and mannose in a molar ratio of 1:1.

### 3.6. Essential Oils

Essential oils are present in some species of *Dipsacus* and *Scabiosa* (Table 7) [35,83,102].

The essential oils isolated by hydrodistillation from dried and fresh roots and leaves of *D. fullonum* were rich in many components; however, quantitative and qualitative differences were noted. The dominant compound in the essential oils from leaves, regardless of fresh or dried materials, was phytol (branched-chain unsaturated diterpene alcohol; precursor for vitamins E and K1), whose content ranged from 61.08% to 72.31%. It was not detected in root essential oil. In addition, the main components in fresh leaf essential oil, i.e., with a level above 5%, were 9,12,15-octadecatrienoic acid methyl ester and cyclohexane, cyclopropylidene. On the other hand, in the essential oils from the dry and fresh roots, the main component was *n*-hexadecanoic acid, especially in the dried material (16.00%), which was also rich in 11,14,17-eicosatrienoic acid, methyl ester (15.86%). *n*-hexadecanoic acid was also identified in the essential oils from dried and fresh leaves but at much lower levels (2.01–2.34%) [35]. In the essential oil from the flowering aerial parts of D. *japonicus*, linalool (11.78%), *trans*-geraniol (8.58%), and 1,8-cineole (7.91%) predominated. The other main ingredients present at above 5% were *β*-caryophyllene (5.58%), *α*-terpineol (5.32%), *β*-selinene (5.15%), and spathulenol (5.04%) [102].

Qualitative and quantitative differences were also observed in the essential oils isolated from different parts of *S. arenaria* [83]. The main compounds detected in the oil from the aerial parts and flower oils were chrysanthenone (23.43–38.52%), camphor (11.75–12.98%), and α-thujone (9.5–10.7%), while α-thujone (34.39%), camphor (17.48%), and β-thujone (15.29%) predominated in the fruit oil. In addition, longifelone (2.41–3.96%) and filifolone (1.99–3.72%) were only identified in oils from the vegetative parts of the plants and the flowers. In *S. atropurpurea* stems, in volatile fractions VF1 (extracted by hexane) and VF2 (extracted by chloroform), the most abundant ingredients were 1,8 cineole (8.1–33.8%), tetradecene (5.7–24.1%), and (E)-β-ionone (5.9–20.7%). It is worth mentioning that dihydroactinidiolide, which was present in significant amounts (26.1%), was identified only in the chloroform fraction [46].

**Table 7:** The main chemical constituents of the essential oils isolated from some species of *Dipsacus* and *Scabiosa*.

Compounds (Content)	Species (Part of Plant)
n-hexadecanoic acid (2.92–16.00%); 11,14,17-eicosatrienoic acid, methyl ester (15.86% only in dried material); 9,12,15-octadecatrienoic acid, methyl ester (0.65–3.2%)	*D. fullonum* (roots; fresh or dried) [35]
Phytol (61.08–72.31%); 9,12,15-octadecatrienoic acid, methyl ester (6.06% only in fresh material); cyclohexane, cyclopropylidene (2.90–5.20%); n-hexadecanoic acid (2.01–2.34%); 3-buten-2-one, 4-(2,6,6-trimethyl-1-cyclohexen-1-yl) (1.5–3.2%)	*D. fullonum* (leaves; fresh or dried) [35]
Linalool (11.78%); *trans*-geraniol (8.58%); 1,8-cineole (7.91%); *β*-caryophyllene (5.58%); *α*-terpineol (5.32%); *β*-selinene (5.15%); and spathulenol (5.04%); geranyl acetone (3.88%); *α*-pinene (3.57%)	*D. japonicus* (flowering aerial parts) [102]
1,8 cineole (8.1–33.8%); tetradecene (5.7–24.1%); (*E*)-β-ionone (5.9–20.7%); dihydroactinidiolide (26.1% in chloroform fraction, not detected in hexane fraction); (Z)-jasmone (5.6% in hexane fraction, not detected in chloroform fraction); eugenol (3.6% in hexane fraction, not detected in chloroform fraction); (*E*)-β-damascenone (3.0–6.4%); (E)-geranylacetone (3.0–9.2%); linalool (3.3–4.9%); 2-hydroxy-5-methylacetophenone (2.9–4.4%); cis-linalool oxide (1.6–3.0%)	*S. atropurpurea* (stems; volatile fraction extracted by hexane or chloroform) [46]
Chrysanthenone (23.43%), camphor (12.98%) and α-thujone (10.7%); α-fenchol (4.08%); sabinene (3.11%); trans-alloocimene (3.03%);	*S. arenaria* (vegetative parts; stems and leaves) [83]
Chrysanthenone (38.52%), camphor (11.7%) and α-thujone (9.5%); α-fenchol (5.86%); filifolone (3.72%); longifolene (3.96%)	*S. arenaria* (flowers) [83]
α-thujone (34.39%), camphor (17.48%), and β-thujone (15.29%); camphene (3.62%); 1,8-cineole (3.48%); sabinene (3.46%)	*S. arenaria* (fruits) [83]

### 3.7. Fatty Acids

The presence of fatty acids has only been investigated in *S. stellata* aerial parts and *D. asper* roots [6,49] (Table 8). Thirteen fatty acids including saturated and unsaturated acids have been identified. The fatty acids present in the hexane extract of *S. stellata* aerial parts accounted for 87% of the total content with linolenic acid, palmitic acids, and linoleic acids predominating [49].

**Table 8:** Fatty acids identified in *Dipsacus* and *Scabiosa* species.

No.	Compound Name	Species (Part of Plant)
1.	Behenic acid	*S. stellata* (aerial parts) [49]
2.	Dodecanoic acid	*S. stellata* (aerial parts) [49]
3.	Dotriacontanic acid	*D. asper* [6]
4.	Eicosanoic acid	*S. stellata* (aerial parts) [49]
5.	Hexadecatrienoic acid	*S. stellata* (aerial parts) [49]
6.	Lignoceric acid	*S. stellata* (aerial parts) [49]
7.	Linoleic acid	*S. stellata* (aerial parts) [49]
8.	Linolenic acid	*S. stellata* (aerial parts) [49]
9.	Myristic acid	*S. stellata* (aerial parts) [49]
10.	Palmitic acid	*S. stellata* (aerial parts) [49]
11.	Pentacosanoic acid	*D. asper* (roots) [6]
12.	Stearic acid	*S. stellata* (aerial parts) [49] *S. tschilliensis* (flowers) [94]
13.	Triacontanoic acid	*S. stellata* (aerial parts) [49]

## 4. Safety of Use

The use of traditional medicines, herbs, and supplements of plant origin is becoming more popular. However, it is necessary to determine the side effects that they may cause or their toxicity to use safely.

An in vitro study showed that the extract of *D. asperoides* was not toxic for normal cells at a concentration up to 500 µg/mL [26,112]. The viability of RAW 264.7 macrophages and periodontal ligament stem cells after treatment with water or ethanol extracts for 24 h was about 90–100% [26,112]. However, the periodontal ligament stem cells treated for 7–21 days with 500 µg/mL of *D. asper* ethanol extract showed morphology changes [112]. The cell viability of J774A.1 murine macrophage was also not affected by the methanol extract of *D. inermis* at a concentration up to 100 µg/mL [39]. The concentration above 300 µg/mL decreased the cell viability below 80%. In addition, *Dipsaci radix* stimulated the proliferation of MC3T3-E1 and primary osteoblastic cells in the concentration range of 3–300 µg/mL after 24 h and 48 h [113]. It should be also noted that akebia saponin D, a quality indicator of *Dipsaci radix*, at a concentration of 25–200 µM has no cytotoxic effect in mouse primary chondrocytes after 24 h [25]. In addition, saponin favored the proliferation of rat bone marrow stomal cells on days 4 and 7 in a dose-dependent manner (0.01–10 µM) [64] and enhanced the proliferation of human mesenchymal stem cells at a concentration up to 1 mg/L after 3 and 5 days [114].

The toxicity of *Dipsacus* or *Scabiosa* plants has been tested not only in vitro but also in vivo. According to Zhou et al. [115], the clinical safety of *D. asper* has been evaluated by Zhan et al. [116]. The total saponin extract (0.28 g/tablet) was administered to volunteers for six months, resulting in only mild side effects such as abdominal discomfort, constipation, swollen gums, raised level of blood alanine aminotransferase (ALT), and dysphoria.

A study using F344 rats which were orally treated with the aqueous extract of *Dipsaci radix* at doses of 0.125, 0.25, 0.5, 1, or 2 g/kg body weight (b.w.)/day for 13 weeks resulted in no deaths or pathophysiological changes [20]. On the other hand, Xiao et al. [117] found that an extract of *D. asper* roots may have an adverse effect at a concentration of 2–32 g/kg/day. *Dipsaci radix* enhanced fetal malformation in a dose-dependent manner in pregnant ICR mice. In addition, the extract at a dosage of 32 g/kg/day, i.e., 17-times higher than recommended for an adult human, was toxic to the fetus; it led to abnormalities of fetal skeletal development including malformed limbs (polydactylia) and sternum (hypoplasia and split) as well as inhibited mineralization cartilaginous tissue and osteogenesis. It is worth emphasizing that the extract also inhibited the mouse embryogenic stem cells and 3T3 cells growth in a dose-dependent manner (0.1–125 mg/mL) with IC_50_ values of 6.83 mg/mL and 5.13 mg/mL, respectively [117].

The ethyl acetate extract of *S. stellata* whole plants orally administered for 14 days at a concentration of 0.5–2 g/kg in a single dose was not toxic for albino Wistar rats and did not induce animal mortality [50]. Moreover, no changes in respiration and urination or in hematological and serum biochemical parameters such as ALT, aspartate aminotransferase (AST), total bilirubin, urea, creatinine, cholesterol, triglycerides, and glucose levels were observed in comparison to those in the control animals [50]. Mouffouk et al. [77] estimated the cytotoxicity of the hydroethanolic extract, petroleum ether, ethyl acetate, and *n*-butanolic fractions of *S. stellata* whole plants in larvae of brine shrimp lethality method. A dose-dependent pattern (at a concentration of 10–100 µg/mL) in the mortality rate of the brine shrimp nauplii was noted. Only the *n*-butanolic extract caused mortality above 50% at a concentration of above 80 µg/mL. The 70% ethanol extract of aerial parts of *S. atropurpurea* possessed an LD_50_ value for adult male albino rats of 5 g/kg b.w. [43].

## 5. Pharmacokinetic and Bioavailability of Some Specialized Metabolites of *Dipsaci radix*

The synergistic effect of numerous, various specialized metabolites of plants is responsible for the medicinal properties of plants. Therefore, knowing the chemical composition of herbal materials is the basis for understanding the mechanisms of their action [118]. It is important to know the pharmacokinetics of drugs to understand the toxicity of their preparations or their therapeutic potential. The therapeutic effectiveness of the preparations/compounds is related to their bioavailability. The bioavailability of compounds depends on many different parameters, such as digestion, absorption, or metabolism [119]. Akebia saponin D, the main ingredient of *Dipsaci radix*, shows low absorption. Therefore, its therapeutic effect is limited [119]. Wang et al. [119] suggested that the microcrystalline form of akebia saponin D obtained by antisolvent precipitation may enhance the bioavailability of akebia saponin D.

A few specialized metabolites identified in the roots of *D. asper*, e.g., 4-*O*-caffeoylquinic acid, 3-*O*-caffeoylquinic acid, 3,5-di-*O*-caffeoylquinic acid, loganic acid, loganin, sweroside, dipsacoside B, and asperosaponin VI, showed rapid absorption after intragastric administration to Sprague-Dawley rats at a concentration of 75.6 g/kg; these ingredients reached the maximum plasma concentration in an hour [15]. In addition, it was shown that sauteing with rice wine of *D. asper* roots enhanced the bioavailability of these specialized metabolites, indicated by a significant increase in maximum plasma concentration and area under the curve for the plasma concentration from zero to the last quantifiable time-point as well as an increase in the level of bioactive compounds in rat liver and kidney tissues compared with those in the crude material (aqueous extract) [15,120].

## 6. Biological Activities of *Dipsacus* and *Scabiosa* Species

### 6.1. Strengthening the Bone Tissue and Antiarthritic Activity

The roots of *D. asperoides*, known in Chinese as Xu Duan, means “connects broken bones” [26]. Until now, numerous in vitro and in vivo studies have demonstrated that *Dipsaci radix* or the pure compounds isolated from this plant material can be potential agents for promoting osteoblast formation and may have an anabolic systemic skeletal effect. *Dipsaci radix* can improve bone density and affect bone histomorphology. *Dipsaci radix* has also shown osteoprotective properties in ovariectomized animals [6,18,23,25,68,110,111,112,113,115,121,122,123,124,125,126,127,128]. In healthy BALB/c mice, an increase in the bone trabeculae density, bone volume/tissue volume ratio, bone surface/tissue volume, and trabecular number and the depletion in the trabecular separation on the proximal tibia after drinking water extract of *Dipsaci radix* were observed [61].

Osteoporosis is characterized by an increase in bone fragility. Osteoporosis can usually occur with aging and after menopause due to estrogen deficiency, which contributes to the reduction of bone density and bone mass and degradation of the microstructure [129]. The hormone replacement therapy that is used in osteoporosis treatment increases bone density. However, the use of hormone replacement therapy for a long time should be limited due to the serious side effects of its use [126]. Modern therapies enhance bone metabolism by promoting osteoblast activity and by inhibiting the effects of osteoclasts [126]. The ethanol extract of *D. asperoides* roots showed concentration-dependent progestogenic activity (40–100 μg/mL) in the T47D progesterone receptor-positive human mammary adenocarcinoma cell line; 100 μg/mL of extract demonstrated the equivalent of 31.45 ng/mL of progesterone treatment. These results indicate that *D. asperoides* roots can be used as an option for progestins [130]. Moreover, asperosaponin D may promote the osteogenic differentiation of human mesenchymal stem cells through the estrogen signaling pathway [131].

Liu et al. [18,122] reported that *Dipsaci radix* decoction has anti-osteoporosis properties in ovariectomized Wistar or Sprague-Dawley rats (oral treatment at a dose of 100–500 mg/kg b.w./day) by increasing trabecular bone formation and bone mineral density and preventing bone mass loss and trabecular structure changes; it also decreases the serum alkaline phosphatase (ALP) level and the level of bone turnover markers, e.g., serum osteocalcin and urinary deoxypyridinoline/creatinine ratio, and receptor activator for nuclear factor κB ligand (RANKL) in osteoblasts and bone marrow stromal cells of the tibia. A recent study showed that *Dipsaci radix* may be able to control osteoblast differentiation, osteoclast proliferation, and mineralization via regulating mitogen-activated protein kinases (MAPK), nuclear-kappa B factor (NF-κb), TNF-α, and Toll-like receptor (TLR4) signaling pathways [127]. Intragastric treatment of bilaterally ovariectomized Wistar rats with wine processed *Dipsaci radix* (with a dose of 75.6 g/kg/day) resulted in protection from an increase in urine Ca/creatinine and P/creatinine levels and serum ALP and osteocalcin concentrations and increased the femur bone mineral density. These effects were comparable to that observed in rats treated with 17*β*-estradiol [68].

In traditional Chinese medicine, Xian-Ling-Gu-Bao capsules have been used to prevent and treat osteoporosis, osteoarthritis, aseptic bone necrosis, or climacteric syndrome. Xian-Ling-Gu-Bao was officially approved in 2002 by the China Food and Drug Administration as an over-the-counter drug for the treatment of osteoporosis [132,133]. This product is composed of the raw material of six plant species: *Epimedii herba* (70%), *Dipsaci asperoidis radix* (10%), *Anemarrhenae rhizoma* (5%), *Psoraleae fructus* (5%), *Rehmanniae radix* (5%), and *Salviae miltiorrhizae radix* (5%) [133]. Xian-Ling-Gu-Bao administered to ovariectomized C57/BL6 mice for six weeks displayed anti-osteoporosis effects by enhancing bone mineral density and bone strength and by decreasing the serum level of the bone formation marker procollagen type I N-terminal propeptide (PINP) and the bone resorption marker C-terminal telopeptide of type I collagen (CTX) [125]. Wu et al. [132] also reported that Xian-Ling-Gu-Bao showed the ability to prevent osteoporosis in two osteoporosis models, prednisolone-treated zebrafish (*Danio rerio*) and ovariectomized Sprague-Dawley rats. Xian-Ling-Gu-Bao altered the protein levels of osteoprotegerin and RANKL. An increase in the OPG/RANKL ratio may suppress bone loss. It is worth mentioning that a dose of 1800 mg/kg (a concentration of six-times the recommended daily dose) did not cause toxicity or adverse effects in the heart, kidney, liver, stomach, or small intestine [132].

Another formulation containing *Dipsaci radix* used in traditional Chinese medicine for treating kidney disease, osteoporosis, strengthening bones, and bone fractures is Du-Zhong-Wan. This preparation is composed of *Eucommiae cortex* (salted bark of *Eucommia ulmoides* Oliv.) and *Dipsaci radix* in an equal weight ratio (1:1) [23]. It was shown that Du-Zhong-Wan displayed anti-osteoporotic activity in the Sprague-Dawley rat osteopenia model. After treatment with Du-Zhong-Wan at a dose of 2–6 g/kg/day for 12 weeks, an increase in the bone mineral density of the femur was observed, together with an improvement of trabecular bone mass and microarchitecture, reduction of the bone resorption and tartrate-resistant acid phosphatase 5b (TRACP-5b) level, and higher serum level of osteocalein, and serum and endometrium level of estrogen [23]. Tian et al. [134] reported that Du-Zhong-Wan favored fractured callus formation and improved osteoblastogenesis and angiogenesis by increasing the level of the H-type vessel endothelium markers (CD31 and endomucin) and proangiogenic factor SLIT3 in C57BL/6 mice after ovariectomy with the open transverse femoral fracture.

Niu et al. [113,123] showed that the 60% ethanolic extract and total saponins of *Dipsaci radix* at a concentration of 50–500 mg/kg/day may have anti-osteoporotic properties in Sprague-Dawley female rats after bilateral ovariectomy or hindlimb unloading Sprague-Dawley rat model by preventing a decrease in bone mass and by improving bone mineral density, biomechanical strength, and trabecular bone architecture. In addition, *Dipsaci radix* suppressed osteoclastogenesis through a reduction of the serum level of bone turnover marker (osteocalcin) and urine concentration of phosphorus, calcium, and deoxypyridinoline/creatinine ratio.

In vitro studies demonstrated that *Dipsaci radix* stimulated osteoblastic proliferation, maturation, and differentiation via bone morphogenetic protein-2 (BMP-2)/MAPK/Smad1/5/8-dependent Runt-related transcription factor 2 (Runx2) signaling pathway. *Dipsaci radix* inhibited osteoclastogenesis by an increase in OPG/RANKL ratio in MC3T3-E1 murine preosteoblasts and primary osteoblastic cells [113,124]. The ethanol extract of *D. asper* enhanced osteogenic differentiation of periodontal ligament stem cells through activation of the vascular endothelial growth factor (VEGF)/PI3K/Akt pathway. In addition, periodontal ligament stem cells displayed greater mineralization and mRNA expression of osteogenesis-related genes such as Col-1, ALP, Runx2, and osteocalcin [112].

Treatment with the triterpenoid akebia saponin D also inhibited the osteoclastic gene RANKL in bone marrow mesenchymal stem cells, suppressing osteoclastogenesis and promoting osteogenesis. In addition, the combination of akebia saponin D and BMP-2 immobilized in 2-N, 6-O-sulfated chitosan alleviated osteoclastic formation, enhanced osteogenesis, and promoted angiogenesis by stimulating SMADs, TGF-β1, VEGFA, and OPG/RANKL signaling pathways [135]. Moreover, loganic acid enhanced osteoblastic differentiation in preosteoblast MC3T3-E1 cells and suppressed osteoclast differentiation of primary-cultured monocytes derived from mouse bone marrow [136].

Akebia saponin D was also found to have a potential application in osteoarthritis therapy. Gu et al. [25] demonstrated that it showed anti-inflammatory activity in mouse primary chondrocytes and alleviated osteoarthritis following the surgical destabilization of a medial meniscus model of osteoarthritis in C57BL/6 male wild-type mice. Akebia saponin D was able to inhibit the production of cyclooxygenase-2 (COX-2), inducible nitric oxide synthase (iNOS), nitric oxide (NO), prostaglandin E2 (PGE2), IL-6, TNF-α, and NF-κB; it also activated the nuclear factor erythroid-2-related factor 2 (Nrf2)/Heme oxygenase 1 (HO-1) pathway and suppressed the expression of matrix synthesis degradation-related proteins such as disintegrin, metalloproteinase with thrombospondin motifs 5 (ADAMTS-5), as well as MMP13 in IL-1β treated chondrocytes. It was also found to enhance the expression of Aggrecan and Collagen II [25]. A recent study found it to also enhance the proliferation and differentiation of human mesenchymal stem cells into nucleus pulposus-like cells through p-ERK1/2 and p-Smad2/3 activation, which may prevent intervertebral disc degeneration [114]. Akebia saponin D also favored the proliferation and osteogenic differentiation of rat bone marrow stromal cells through the phosphatidylinositol-3 kinase/AKT signaling pathway, thus inhibiting osteoporosis. This active compound elevated osteogenic differentiation markers such as ALP activity and calcium deposit formation and mRNA level of osteogenic-related genes (ALP, osteocalcin, type 1 collagen (COL 1), and RUNX2) [64].

Another terpenoid compound, hederagenin, also alleviated the progression of osteoarthritis and inhibited inflammation and cartilage degradation. In an in vitro study, hederagenin exerted chondroprotective and anti-inflammatory effects by suppressing the JAK2/STAT3/MAPK pathway, inhibited extracellular matrix degradation, elevated Aggrecan and Collagen II levels, and reduced levels of MMPs and ADAMTS5. In addition, this pentacyclic triterpenoid saponin inhibited cartilage destruction in rats induced by monosodium iodoacetate [137]. Hederagenin 3-*O*-(2-*O*-acetyl)-α-l-arabinopyranoside and the dichloromethane fraction of *Dipsaci radix* also improved osteoblastic differentiation of human alveolar bone marrow-derived mesenchymal stem cells. This specialized metabolite caused the formation of calcified nodules and enhanced the level of bone differentiation protein expression such as sialoprotein and osteocalcin similar to dexamethasone [121].

Moreover, the main iridoid glycoside isolated from *Dipsaci radix*, sweroside (at a concentration of 1 µM), enhanced rat osteoblast-like UMR 106 cell proliferation, while loganic acid, loganin, and sweroside favored mineralization. Loganin and sweroside suppressed the formation of adipocytes in 3T3-L1 cells [125]. The anti-osteoporotic property of sweroside was also confirmed by Wu et al. [128]. This compound enhanced mineralization of MC3T3-E1 cells by increasing the protein expression of the membrane estrogen receptor-α and G protein-coupled receptor 30 (GPR30), which activate the p38 signaling pathway.

MC3T3-E1 mouse embryonic osteoblast proliferation and differentiation were also promoted by various concentrations (25, 50, or 100 μg/mL) of a 17 kDa polysaccharide (DAI-1) isolated from *D. asperoides* in high glucose concentrations; this appeared to act via the stimulation of the bone morphogenetic protein 2 (BMP-2)/Smad/runt-related transcription factor 2 (Runx2)/Osterix signaling pathway. The polysaccharide also enhanced osteocalcin level and the mRNA and protein levels of BMP-2 and Runx2 [110]. A homogenous polysaccharide with a molecular weight of 26.1 kDa isolated from the roots of *D. asper* (at a dose of 50 or 200 mg/kg b.w.) was also able to enhance mRNA and protein levels of VEGF and osteoprotegin, suppress mRNA and protein expression levels of RANKL, and activate the PI3K/Akt/eNOS signaling pathway in ovariectomized rats [111]. The water-soluble polysaccharide (ADAPW), with an average molecular weight of 16 kDa, inhibited the viability of the human osteosarcoma HOS cells by induction of apoptosis cells and inhibition of the PI3K/Akt signaling pathway [11].

An increase in bone resorption may also lead to osteo- or rheumatoid arthritis. Rheumatoid arthritis is a chronic autoimmune and inflammatory disease of the connective tissue [17]. *Dipsaci radix* has been also applied for many years in traditional Chinese medicine to treat other bone diseases such as rheumatic arthritis [6]. The aqueous extract of *D. asperoides* roots at a concentration of 50 mg/kg and 100 mg/kg, administered orally once a day for 21 days, displayed antiarthritic effects in collagen-induced rheumatoid arthritis in male DBA/1 mice by enhancement of the ankle joint architecture and suppression of arthritis score (synovitis, pannus, and bone erosion scores) and serum levels of anti-CII IgG2a antibody and the inflammatory mediators (TNF-α, IL-1β, and IL-6). These effects were comparable or stronger to those after treatment with 1 mg/kg of the anti-rheumatoid drug indomethacin [138].

Akebia saponin D at a concentration of 10 or 20 mg/kg/day also showed anti-osteoclastogenic activity in the arthritic joints of male BALB/c and DBA/1 with collagen-induced arthritis. The saponin reduced mRNA expression of osteoclastogenesis markers such as TRAP, CtsK, MMP-9, and β3-integrin. In addition, akebia saponin D also inhibited phosphorylation of Akt, p38, and JNK and mRNA and protein levels of osteoclastogenesis markers in RANKL-induced osteoclastogenesis bone marrow-derived monocytes [17]. Dipsaus saponins also inhibited chondrocyte apoptosis in a rat model of osteoarthritis in a dose-dependent pattern by decreasing expression of Bax, caspase-3, and caspase-9 and by increasing expression of Bcl-2 [139]. Cantleyoside, an iridoid identified in *D. asper* roots, inhibited proliferation of human rheumatoid arthritis fibroblast synovial cells (HFLS-RA) and induced cell apoptosis through AMPK/Sirt 1/NF-κB pathway activation [140]. Moreover, the protective effect of sweroside was observed in IL-1β-induced inflammation in rat articular chondrocytes. The anti-inflammatory effect of this iridoid was mediated by the inhibition of NF-κB and mTORC1 signaling pathways [141].

These above findings suggest that *Dipsaci radix* may have a beneficial therapeutic effect in the treatment of postmenopausal osteoporosis and may protect against arthritis.

### 6.2. Anti-Neurodegenerative Activity

Alzheimer’s disease is a neurodegenerative disease that destroys memory and deteriorates cognitive function [142].

Five-month administration of 4 g/kg ethanol extract of *D. asper* roots improved neurocognitive dysfunction in the passive avoidance task and diminished expression of hippocampal β-amyloid protein (Aβ) positive cells in aluminum chloride-treated male Sprague-Dawley rats. This effect increased with the time of treatment (1–5 months). It may be important in the treatment of Alzheimer’s disease and memory system dysfunction [31]. Akebia saponin D, at a dose of 30–270 mg/kg administered for four weeks, also showed a preventive effect against memory cognitive impairment in ibotenic acid-exposed male Sprague-Dawley rats [143]. Moreover, it was shown that the saponin can protect against learning and memory dysfunction in rats induced by bilateral intracerebroventricular injections of Aβ1–42 in the Y-maze and Morris water-maze tests. Akebia saponin D attenuated the activation of microglia and astroglia and protein expression level of IL-1, COX-2, TNF-α, NF-κB, and Akt phosphorylation in the rat brain [143]. The total saponin of *D. asperoides* also possessed neuroprotective properties in rat cortical and hippocampal neurons against damage induced with β-amyloid protein, ameliorated cell viability, and decreased lactate dehydrogenase (LDH) release and lipid peroxidation at a concentration of 150–300 mg/L [144]. Other triterpene compounds, such as oleanolic acid, ursolic acid, and hederagenin, also showed neuroprotective activity in multiple brain disorders [145,146]. For example, hederagenin diminished Aβ deposition in the head area of *Caenorhabditis elegans*, ameliorated cognitive impairment or pathological changes in APP/PS1 mice, and induced PPARα/TFEB-dependent autophagy of BV2 cells [145]. Moreover, treatment with sweroside, a secoiridoid glycoside, alleviated memory deficits in scopolamine-induced Zebrafish (*Danio rerio*) in behavioral tests such as the tank diving test, the Y-maze, and the novel object recognition test [147]. The flavonoid apigenin also suppressed neurotoxicity and cognitive function in LPS-induced mice. The compound protected against neuronal degenerative changes in mice hippocampi [148].

It was also found that 50% methanol extracts from leaves and roots of *D. fullonum* demonstrated anti-acetylcholinesterase activity [33]. The ethyl acetate and *n*-butanolic fractions of 70% methanol extract from *S. stellata* whole plants, at a concentration of 200 μg/mL, exhibited moderate (30.8%) and low (10.9%) acetylcholinesterase (AChE) inhibitory activity, respectively [77]. On the other hand, the ethyl acetate and *n*-butanolic fractions of stems and leaves from *S. arenaria* (at a dose of 1 mg/mL) were able to inhibit AChE in 97.61% and 90.47%, respectively. The value of IC_50_ ranged from 0.016 mg/mL to 0.029 mg/mL. In comparison to eserine (positive control), the IC_50_ value was 0.0029 µg/mL [72]. The root extract also displayed potent activity. The strongest effect was demonstrated by the *n*-butanolic fraction (87.61% AChE inhibition and IC_50_ value = 0.02 mg/mL) [73]. Yu et al. [96] showed the effectiveness of some compounds isolated from the roots of *D. asper*; for example, oleanane triterpenoid saponin (3-*O*-*β*-d-glucopyranosyl-(1→3)-α-l-rhamnopyranosyl-(1→2)-α-l-arabinopyranosyl-23-hydroxyolean-18-en-28-oic acid 28-*O*-*β*-d-glucopyranosyl-(1→6)-*β*-d-glucopyranosyl ester) was found to inhibit acetylcholinesterase with an IC_50_ value of 15.8 μM. Other terpenoid compounds isolated from *Dipsaci radix*, such as dipsacus saponin IV, dipsacus saponin XI, and dipsacus saponin X, displayed strong AChE inhibitory activity, while cauloside A, dipsacus saponin C, and dipsacus saponin XI were more effective against butyrylcholinesterase. These activities were higher than that of the positive control, berberine. Moreover, the saponins were found to be more effective than the iridoids, loganic acid and sweroside. The terpenoids also inhibited β-site amyloid precursor protein cleaving enzyme 1 (BACE1) and advanced glycation end-product (AGE) formation [149].

### 6.3. Hepatoprotective Activity

Liver fibrosis is a chronic liver disease caused by many agents such as hepatitis B virus (HBV), hepatitis C virus (HCV), non-alcoholic steatohepatitis, or alcoholic fatty liver disease [150].

The flavonoids and phenolic acids from *Scabiosa* spp. have demonstrated anti-hepatic fibrosis potential in male Wistar or male Sprague-Dawley rats treated intraperitoneally with carbon tetrachloride (CCl_4_) (a selective hepatotoxic drug); similar effects were also noted for drugs used in Mongolian medicine, such as Qingganjiuwei and Gurigumu-7 (composed of various herbs including *S. comosa* flowers) [43,47,86,87].

Qingganjiuwei, a drug commonly used in Inner Mongolia in patients with chronic hepatic disease, improved liver morphology and structure in CCl_4_-treated SD rats when administered at 1.575–4.725 g/kg/day for eight weeks. The drug reduced hepatocyte necrosis, lymphocytic infiltration, and pseudolobuli and lowered COL1, tissue inhibitor of metalloproteinase1 (TIMP1), and α-smooth muscle actin (α-SMA) expression. The drug also activated the MAPK pathway in the liver through the suppression of extracellular signal-regulated kinase (ERK), C-Jun amino-terminal kinases (JNKs), and stress-activated protein kinase-2 (p38 proteins) [86]. Qingganjiuwei also increased mRNA and protein expressions of MMP2 and MMP9 and inhibited the levels of the serum aminotransferases (ALT and AST) [86].

The methanol-eluted fraction of Gurigumu-7 extract (0.264 g/kg) displayed a more potent hepatoprotective effect in mice with CCl_4_-induced liver damage compared to crude Gurigumu-7 extract, even when applied at a four-times higher concentration (1.084 mg/kg). The methanol fraction alleviated histopathological changes in the liver and serum ALT, ASP, and liver malonyldialdehyde (MDA) levels and enhanced the liver superoxide dismutase (SOD) level in a dose-dependent manner (66, 132, and 264 mg/kg) [87].

Anti-hepatic fibrosis was also reported for *S. comosa* and *S. tschilliensis* [79,80]. Some specialized metabolites inhibited the viability of hepatic stellate LX-2 cells at concentrations of 12.5–200 μM; this included those belonging to flavonoids, which accounted for about 60% of the total identified compounds in the inflorescences of *Scabiosa* plants. The flavonoids enhanced the expression of Stat1, Pparg, Hsp90aa1 genes, signal transduction and transcriptional activator 1 (STAT1), and peroxisome proliferator-activated receptor G (PPARG) proteins, which play key roles in the pathogenesis of liver fibrosis [79]. Apigenin exhibited the strongest ability to inhibit cell proliferation [79]. The flavonoid-rich extract of *S. comosa* inflorescences at concentrations of 100 and 200 mg/kg also suppressed hepatic fibrosis in Wistar rats pre-treated with CCl_4_; the extract inhibited the level of biochemical parameters in blood serum (ALT, AST, ALP, and hyaluronic acid), the markers of liver fibrosis (laminin, amino-terminal propeptide of type III procollagen (PIIINP), collagen IV, collagen deposition in the liver tissues), and expression of α-SMA, collagen I, and fibronectin [47]. Moreover, the extract attenuated phosphorylation of Smad3 in liver tissue and TGF-β1-pre-treatment primary mouse hepatic stellate cells. In the latter, it was observed that the expression of the fibrotic genes (α-SMA, collagen I, and fibronectin) was suppressed in a dose-dependent manner [47]. Ethanol extract of aerial parts of *S. atropurpurea* and hexane, ethyl acetate, *n*-butanolic, and chloroform fractions were able to decrease the levels of serum ALT, AST, and ALP in CCl_4_-induced liver damage in albino rats after treatment with a dose of 100 mg/kg [43].

Sweroside demonstrated a protective effect against liver fibrosis in mouse models treated with CCl_4_ and methionine-choline-deficient diet-induced non-alcoholic steatohepatitis [151,152]. Sweroside treatment yielded an anti-fibrotic effect through the FXR-miR29a pathway [151]. Sweroside also improved NASH symptoms by inhibiting the activation of the hepatic NLRP3 inflammasome [152]. Apigenin was found to inhibit palmitic acid-induced pyroptosis by regulating the pyrin domain containing 3 (NLRP3) inflammasome in HepG2 cells and primary mouse hepatic cells [153]. Other polyphenols, derivatives of caffeoylquinic acid such as 1,5-di-*O*-caffeoylquinic acid, isochlorogenic acid C, isochlorogenic acid B, chlorogenic acid, isochlorogenic acid A, neochlorogenic acid, and caffeic acid also suppressed LX-2 hepatic stellate cell growth [79]. The two predominant caffeoylquinic acid derivatives in inflorescences of *S. comosa* and *S. tschilliensis*, viz., 3,5-di-*O*-caffeoylquinic acid and chlorogenic acid, demonstrated anti-hepatitis C virus (HCV) activity in the Huh-7.5 cell line infected with HCV [74].

In addition, some triterpenoid derivatives isolated from the whole plants of *S. tschiliensis*, such as scabiosaponins E, F, G, I, and J; hookerosides A and B; and prosapogenin 1b, showed strong inhibition of pancreatic lipase. Moreover, 0.12 mg/mL prosapogenin 1b exerted similar inhibitory properties as the lipase inhibitor orlistat at a concentration of 0.005 mg/mL [51]. Akebia saponin D reduced lipid droplet accumulation in BRL cells, attenuated hepatic steatosis, and elevated the expression of Bcl-2/adenovirus E1B 19-kDa interacting protein 3 (BNip3) and phospho-AMPP; it also improved mitochondrial function and autophagy modulation, inhibited rotenone-induced BRL cell apoptosis, elevated Bcl-2/Bax ratio, and suppressed the level of intracellular reactive oxygen species (ROS) and mitochondrial membrane potential loss in rotenone-treated BRL cells and rat liver mitochondria [154,155].

### 6.4. Cardioprotective Activity

Cardiovascular diseases are one of the leading causes of death worldwide [156].

The anti-atherosclerotic effect of akebia saponin D was studied in vitro in H_2_O_2_-treated human umbilical vein endothelial cells (HUVECs) and in vivo in *ApoE*^−/−^ mice [157]. It was shown that this saponin protected against H_2_O_2_-induced cytotoxicity in HUVEC, inhibited ROS level, the mitochondrial membrane potential disruption, and apoptosis in oxidative stress-induced endothelial cells in a dose-dependent manner (50–200 µM); the mechanism was believed to involve increasing Bcl-2 family protein levels and decreasing caspase-3 and Bax activation. Moreover, doses of 150 mg/kg/day and 450 mg/kg/day reduced aortic plaque formation in mice as well as aortic and liver apoptosis, serum triglyceride (TG), total cholesterol (TC), low-density lipoprotein cholesterol (LDL-C), and lipid deposition in the liver, as well as atherosclerotic lesion size; it also enhanced the expression of antioxidant enzymes (SOD, catalase (CAT), and glutathione (GSH)) in vascular tissue and liver [157]. Li et al. [158] demonstrated that sweroside has a protective effect on ischemia-reperfusion-induced myocardial injury by inhibiting oxidative stress and pyroptosis partially via modulation of the Kelch-like ECH-associated protein 1 (Keap1)/Nrf2 pathway. The iridoid also suppressed aconitine-induced cardiac toxicity in the H9c2 cardiomyoblast cell line [159]. Long-term gavage (for six weeks) of akebia saponin D protected against fibrosis myocardial ischemia injury, inhibited cardiac dysfunction, and reduced infarct size in a Sprague-Dawley rat model with chronic myocardial infarction induced by permanent ligation of the left coronary artery. Treatment with this saponin decreased hydroxyproline level and changed the activity of the oxidative stress enzymes by elevating SOD and DSH-peroxidase (GSH-Px) levels and by reducing MDA content. Moreover, akebia saponin D regulated inflammatory mediators by diminishing the levels of TNF-a and IL-6 and by elevating the level of IL-10 [160]. Several in vitro and in vivo studies found flavonoids detected in *Scabiosa* spp., such as apigenin and luteolin, to have cardioprotective properties [161,162,163].

However, Song et al. [164] demonstrated that *D. asper* roots, drunk widely as a tea for beneficial health effects, or dipsacus saponin D may have an undesirable effect on platelets and may increase the risk of thrombosis. *D. asper* roots were found to be an herb with procoagulant activities on platelets and prothrombotic properties. Dipsacus saponin C elevated procoagulant activity in a dose- and time-dependent manner, elevated intracellular calcium level, and decreased ATP. In addition, it caused translocation of Bax and Bak, cytochrome c release, caspase-3 activation, and the disruption of mitochondrial membrane potential. The oral administration of 10 mg/kg and 25 mg/kg dipsacus saponin C also resulted in an increase in thrombus formation in a rat venous thrombosis model [164].

### 6.5. Renal and Gastritis Protection

A 61 kDa polysaccharide (WDRAP-1) isolated from *D. asperoides* roots showed protective activity against oxidative stress generated in renal ischemia-reperfusion injury in male Wistar rats and displayed strong superoxide and hydroxyl radical scavenging activities in vitro. It was shown that oral pre-treatment of rats with the polysaccharide at a concentration of 50–200 mg/kg b.w. for 14 days before ischemia-reperfusion may improve renal injury (especially at the highest dose); treatment suppressed the level of renal injury indicators including creatinine, blood urea nitrogen, lactate dehydrogenase (LDH), and serum MDA and enhanced serum SOD and some renal tissue antioxidant enzyme activities (SOD, GSH-Px, and CAT) [63]. Hederagenin was also found to protect against renal fibrosis. This terpenoid compound attenuated the proliferation and fibrosis of TGF-β-treated NRK-49 F cells by targeting the muscarinic acetylcholine receptor [165].

Dipsacus saponin C, a saponin isolated from the roots of *D. asper*, was found to have a protective effect against HCl·ethanol-induced gastritis and indomethacin-induced gastric ulcers in male Sprague-Dawley rats. It was shown that treatment with dipsacus saponin C caused a decrease in gastric secretion volume and gastric acid production in pylorus-ligated rats. It was found that this compound had a moderate effect on colonization and growth inhibition of *Helicobacter pylori* at a concentration of 50–100 µM. In addition, dipsacus saponin C also showed a cytotoxic effect for SNU638 and AGS human gastric cancer cells with an IC_50_ at 54.6 mM and 37.3 mM, respectively [91].

Moreover, akebia saponin D may exert a therapeutic role by regulating the intestinal microbiome and protecting intestinal epithelial cells from external damage. It was found to achieve this by inhibiting oxidative damage to the intestinal barrier by downregulating PPAR-γ/FABP4 in the human intestinal cell line FHs74 Int [166].

Luteolin was also found to have therapeutic effects against interstitial fibrosis-induced renal anemia in vitro and in vivo. This activity was mediated via the SIRT1/forkhead box O3 (FOXO3) pathway [167].

### 6.6. Anti-Asthmatic Effect

*Dipsaci radix* alleviated the asthmatic response in BALB/c mice with allergic asthma induced by an ovalbumin. *Dipsaci radix* treatment at a dose of 20 m/kg or 40 mg/kg resulted in attenuation of the methacholine response, inflammatory cell infiltration, and mucus secretion in the bronchial airway and a decrease in the levels of pro-inflammatory cytokines (IL-5 and IL-13) in bronchoalveolar lavage fluid, eotaxin, serum total IgE, expression of iNOS, and NF-κB phosphorylation in lungs [27]. Similarly, apigenin, one of the flavonoids identified in *Scabiosa* spp., was found to inhibit inflammatory mediators and eosinophilia in lung and airway tissues in in vivo acute lung injury and asthma models [163].

### 6.7. Anti-Diabetic Activity

A polysaccharide (DAP) isolated from *D. asper* roots demonstrated beneficial effects on renal function and renal pathological changes as well as antihyperglycemic, hypolipidemic, and antioxidant activities in streptozotocin-induced diabetic Wistar rats [16]. Four weeks of intragastric administration (100 mg/kg and 300 mg/kg per day) in type 2 diabetic rat model resulted in a reduction of glycosylated, fasting blood glucose, serum creatinine, blood urea nitrogen, urine protein, and urinary albumin excretion. Moreover, oral administration of the polysaccharide (300 mg/kg) suppressed the serum level of TC, TG, LDL, and renal AGE-RAGE formation (advanced glycation end products-receptor for advanced glycation end products) and enhanced SOD, CAT, and GSH activities in the kidney of rats with diabetic nephropathy [16]. Similar antilipemic effects were also observed for hederagenin. This pentacyclic triterpene exerts its potential through the p38MAPK pathway in oleic acid-induced HepG2 cells and in hyperlipidemic Sprague-Dawley rats [168].

The ethanol extract and the hexane, ethyl acetate, *n*-butanolic, and chloroform fractions of the aerial parts of *S. atropurpurea* demonstrated anti-hyperglycemic activity by decreasing the blood glucose level in albino rats with alloxan-induced hyperglycemia [43]. The methanol extract of *S. atropurpurea* subsp. *maritima* whole plants demonstrated α-glucosidase inhibitory activity with an IC_50_ value = 100 μg/mL. This effect was higher than that found for the positive control, the anti-diabetic drug acarbose (IC_50_ = 196 μg/mL) [78]. Methanolic extracts from the fresh leaves and roots of *D. fullonum* also inhibited porcine pancreatic α-amylase activity [35]. These extracts demonstrated low effectiveness in this study, with the strongest activity being found to be IC_50_ = 86.01 µg/mL for the dried leaf extract. This activity was more than 100-times lower compared to that of acarbose (IC_50_ = 0.69 μg/mL).

### 6.8. Anti-Inflammatory Activity

Several plant species of *Dipsacus* and *Scabiosa* or some specialized metabolites isolated from them can be valuable, new anti-inflammatory agents [26,32,39,50,92,169].

The water extract of *D. asper* roots demonstrated anti-inflammatory properties in the lipopolysaccharide (LPS)-activated murine macrophage cell line RAW 264.7 by suppressing NO production with an IC_50_ = 45.1 µg/mL [92]. In a later study [26], an aqueous extract of *D. asperoides* roots at a dose of 50–500 µg/mL showed inhibitory potential on inflammation and oxidative stress in RAW 264.7 macrophages exposed to LPS; it was found to act by lowering NF-κB and ERK1/2 phosphorylation, nuclear translocation of NF-κB, and activation of Nrf2/HO-1. The extract reduced the levels of inflammatory mediators (iNOS, COX-2, and cytokines IL-6 and IL-1β) as well as ROS levels [26]. The methanol extract of *D. inermis* leaves also showed the ability to inhibit the production of NO, COX-2, PGE2, pro-inflammatory mediators (IL-1β and IL-6, and TNF-α), intracellular ROS level, and phosphorylation of NF-κBp65 and IκBα in a dose-dependent manner (25–100 µg/mL) in the LPS-induced murine macrophage cell line J774A.1 [39]. The anti-inflammatory potential of *D. inermis* leaf extract was also confirmed in vivo in Wistar albino rats; it protected against vascular permeability (caused by acetic acid) and paw oedema (induced by carrageenan) in a concentration- and time-dependent manner. In addition, the serum levels of TNF-α, IL-1β, and IL-6 were significantly reduced while IL-10 level was enhanced after oral administration of the extract at a concentration of 50–100 mg/kg b.w. [39]. Anti-inflammatory properties in a Wistar rat model of carrageenan-induced paw oedema also demonstrated the ethyl acetate extract of *S. stellata* whole plants. The highest activity was observed in the first hour after treatment with the extract at a concentration of 50 mg/kg (72.73% of inhibition). This effect was stronger than that of diclofenac (about 45% of inhibition). In addition, the anti-inflammatory effect of the plant extract lasted up to 24 h [50].

Some compounds belonging to iridoids, saponins, or phenolic acids (for example, dipsasperoside A, dipsanoside A and B, dipsacus saponin A, akebia saponin D, or caffeic acid) isolated from the roots of *D. asper* also were able to reduce the production of NO in RAW 264.7 cells. The potent activity was demonstrated by akebia saponin D and dipasperoside A, with IC_50_ values of 12.7 µM and 15.2 µM, respectively; these values were higher than those for the positive control, a nonselective NOS inhibitor, *N*^G^-monomethyl-L-arginine (IC_50_ = 22.6 µM) [92]. Reduced NO levels and iNOS expression have also been observed in LPS-induced RAW 264.7 cells after treatment with akebia saponin D (at a concentration of 25–100 µM) [32]. Akebia saponin D also suppressed the expression of DNA methyltransferase (DNMT) 3b, the levels of PGE2 and p-STAT3, as well as the protein and mRNA levels of IL-6 and TNF-α [169]. The levels of the inflammatory indicators, prostaglandin E2, i-NOS, COX-2, TNF-α, IL-1β, and IL-6, were also decreased after treatment with 40 and 80 µM sweroside in LPS-induced RAW264.7 cells. In addition, sweroside suppressed inflammation through the sirtulin 1 (SIRT1)/NF-κB and SIRT1/Forkhead transcription factor O1 signaling pathways [170].

Akebia saponin D also showed anti-inflammatory activity in vivo by reducing paw oedema in carrageenan-induced Sprague Dawley rats and by inhibiting xylene-induced ear swelling in mice. It also lowered the level of NO in rat plasma in a carrageenan-induced rat paw oedema model [32].

Anti-inflammatory potential was also demonstrated by apigenin, which was believed to act by the modulation of the p38/MAPK, PI3K/Akt and NF-κB pathways [163].

### 6.9. Antioxidant Activity

The most frequently used methods to determine the antioxidant properties of *Dipsacus* and *Scabiosa* plant extracts were the DPPH (2,2-diphenyl-1-picrylhydrazyl radical scavenging assay), ABTS (2,2′-azinobis(3-ethylbenzothiazoline-6-sulfonic acid) radical cation assay), ORAC (oxygen radical absorbance capacity), FRAP (ferric ion reducing antioxidant assay), and CUPRAC assays (cupric-reducing antioxidant capacity). The antioxidant activity varied depending on the plant material (whole plants, leaves, and roots), the solvent, extraction time, and the extraction method (Table 9) [14,24,33,34,42,46,50,52,53,56,72,73,74,76,77,171,172].

The antioxidant activity of the methanolic extract from *D. asper* roots was confirmed in DPPH and Cu^2+^-mediated LDL oxidation with IC_50_ values of 90.2 and 134.4 µg/mL, respectively. This activity may be attributed to caffeoylquinic acid derivatives identified in root extract such as 3,4-di-*O*-caffeoylquinic acid, 4,5-di-*O*-caffeoylquinic acid, 3,5-di-*O*-caffeoylquinic acid, and their methyl derivatives. These specialized metabolites showed potent antioxidant activity against DPPH radical formation and Cu^2+^-mediated LDL oxidation with IC_50_ values of 10.4–18.2 µM and 1.8–2.3 µM, respectively [24]. The content of the total polyphenols in the acetone/water extract (7:3) of *D. fullonum* whole plant was 19.52 mg GAE (gallic acid equivalents)/g d.w. of plant material; it displayed an antioxidant capacity lower than 5 mmol TEAC (Trolox equivalent antioxidant capacity)/100 g d.w. plant material in the DPPH and ABTS assays [171]. The antioxidant properties were also demonstrated in 50% methanolic extract of leaves and roots (ultrasound assisted extraction) in the ORAC assay with values of 14.78 mmol TEAC/100 g d.w. and 10.87 mmol TEAC/100 g d.w. for the leaf and root extracts, respectively [33]. A similar observation was found by Saar-Reismaa et al. [34]. The crude 70% ethanol extract of *D. fullonum* leaves showed antioxidant activity in the ORAC assay (10.8 mmol TEAC/100 mL). The fraction NP7 of crude leaf extract that was rich in two chlorogenic acid derivatives, saponarin and isoorientin, also displayed antioxidant activity (12.5 mmol TEAC/100 mL), while the fraction NP2 containing *bis*-iridoids was ineffective (0.78 mmol TEAC/100 mL) [34].

On the other hand, the aqueous extract of *D. fullonum* leaves obtained by ultrasound assisted extraction (extraction time, four hours) showed high radical scavenging activity (RSA) (73.81%) in DPPH [172]. The procedure extraction with the aqueous solution of amino acid ionic liquids, viz., triethanolammonium salts of two amino acids methionine ([TEAH]^+^[Met]^−^) and threonine ([TEAH]^+^[Thr]^−^), resulted in a beneficial effect on the antioxidant activity of leaf extracts in FRAP and CUPRAC assays compared to the extract after extraction with the pure water. In addition, extraction with the aqueous solution of [TEAH]^+^[Thr]^−^ (extraction time, two hours) increased the total polyphenol content to 8.16 mg GAE/g raw material. The use of [TEAH]^+^[Met]^−^ in aqueous solution and reducing the extraction time to one hour resulted in a similar level of polyphenol (7.38 mg GAE per g of raw material) [172]. Antioxidant effect was also observed for the extract of *D. sativus* leaf. The extract at a dose of 300 mg/kg/day in ICR mice pre-treated with D-galactose to induce oxidative stress resulted in an increase in the level of SOD and a decrease in the level of MDA in the peripheral blood plasma [42].

*S. stellata* whole plants are a rich source of polyphenolic compounds. The ethyl acetate and *n*-butanolic fractions have moderate antioxidant activity in simple, chemical, antioxidant tests such as DPPH, ABTS assays, and FRAP test. The *n*-butanolic fraction displayed stronger potential in the DPPH assay, i.e., FRS_50_ (free radical scavenge) = 64.46 µg/mL, compared to ascorbic acid FRS_50_ = 8.21 µg/mL [76]. The dichloromethane fraction did not possess significant activity in antioxidant assays, which may be related to the low content (below 1 mg of gallic acid/g dry extract) of phenolic compounds [76]. A 70% ethanol extract of *S. stellata* whole plants in DPPH also displayed antioxidant activity, with an IC_50_ value of 86 μg/mL [75]. The petroleum ether, ethyl acetate, and *n*-butanolic fractions from *S. stellata* whole plants showed various antioxidant potential in different chemical models such as DPPH and ABTS, FRAP, CUPRAC, β-carotene, phosphomolybdate assay, ferrous ions, and metal chelating assays [50]. The strongest effect was demonstrated by the *n*-butanolic fraction in DPPH (IC_50_ = 21.22 μg/mL) and chelation in ferrous iron assay (EC_50_ = 1.65 mg/mL), while the ethyl acetate fraction was most active in ABTS (IC_50_ at 14 μg/mL), CUPRAC (A_0_._50_ = 28.5 µg/mL), and β-carotene assays (IC_50_ = 10.34 μg/mL). In addition, the *n*-butanolic fraction in DPPH had a higher reducing power than butylated hydroxyl toluene (BHT) (22.32 µg/mL) but lower than α-tocopherol (13.02 µg/mL), butylated hydroxy anisole (BHA) (6.82 µg/mL), and ascorbic acid (3.1 µg/mL) [50]. The highest protein denaturation inhibition was found for the ethyl acetate extract, which showed 78.86% inhibition at the maximal tested concentration (1 mg/mL). Ibuprofen (standard drug) at the same concentration caused 100% inhibition [77]. Antioxidant activity was also reported for 70% ethanol extracts of *S. comosa* and *S. tschilliensis* inflorescences. *S. tschilliensis* had a stronger antioxidant activity than *S. comosa* in DPPH, ABTS, and FRAP assays. For example, the IC_50_ values in DPPH were 272.8 µg/mL and 331.1 µg/mL, respectively [74]. Wang et al. [52] reported that the 90% ethanol extract of *S. tschilliensis* at a concentration of 26.5 µg/mL scavenged 50% of DPPH free radicals (IC_50_ value for ascorbic acid was 5.41 µg/mL). The crude extract (95% ethanol) and four solvent partitioned fractions (water, *n*-butanol, ethyl acetate, and petroleum ether) from *S. tschiliensis* whole plants at various growing stages (pre-flowering, flowering, and fruiting stage) showed different antioxidant activities in DPPH, ABTS, inhibition of lipid peroxidation, or OH scavenging activity [53]. The IC_50_ values for the crude extract were in the range of 25.65–86.79 µg/mL while the ethyl acetate fraction from the pre-flowering stage of plants had the highest antioxidant capacity (IC_50_ 8.47 µg/mL) in DPPH. This value was comparable to that of vitamin C (7.6 µg/mL). The ethyl acetate fraction from the pre-flowering stage of plants also possessed the highest ABTS (58.76 µg/mL), hydroxyl radical scavenging ability (67.64 µg/mL), and lipid-peroxidation-inhibition activity [53]. The *n*-butanolic and ethyl acetate fractions of *S. arenaria* roots also displayed strong antioxidant activity in four assays: DPPH, ABTS, reducing power, and β-carotene bleaching inhibition activity. The *n*-butanolic fraction demonstrated excellent ability, mainly in the β-carotene bleaching inhibition assay (IC_50_ = 0.018 mg/mL). This effect was stronger than that obtained for BHT (IC_50_ = 0.04 mg/mL). In the DPPH and ABTS assays, the IC_50_ values for both fractions were comparable to BHT [73]. The ethyl acetate fractions of the roots, flowers, fruits, and aerial parts (stems and leaves) of *S. arenaria* showed the beneficial antioxidant ability in DPPH, with IC_50_ values of 0.017–0.019 mg/mL; the best properties were observed for the flowers [72,73]. Other species of *Scabiosa*, *S. artropurpurea* and *S. atropurpurea* subsp. *maritima*, also demonstrated antioxidant properties. In comparison to ascorbic acid (IC_50_ = 0.084 mg/mL), among four tested extracts, the ethanol extract of *S. artropurpurea* stems exhibited the best antioxidant capacity, with IC_50_ = 0.1383 mg/mL in DPPH assay. In addition, the hexanoic volatile fraction (VF1) and ethyl acetate extract displayed similar effects with IC_50_ values of 0.4798 mg/mL and 0.4806 mg/mL, respectively [46]. The antioxidant activity of 70% ethanol extract of aerial parts of *S. atropurpurea* and hexane, ethyl acetate, *n*-butanol, and chloroform fractions were also demonstrated by increasing blood glutathione in diabetic albino rats [43]. Silver nanoparticles with *S. atropurpurea* subsp. *maritima* water fruit extract also was found as a promising antioxidant agent in DPPH and FRAP assays. The IC_50_ values were 0.112 mg/mL and 0.036 mg ascorbic acid equivalent antioxidant capacity/g d.w., respectively, and were comparable to that for ascorbic acid [56].

The pure compounds isolated from *D. asper* roots, i.e., six dicaffeoylquinic acid derivatives (3,4-di-*O*-caffeoylquinic acid, methyl 3,4-di-*O*-caffeoyl quinate, 3,5-di-*O*-caffeoylquinic acid, methyl 3,5-di-*O*-caffeoyl quinate, 4,5-di-*O*-caffeoylquinic acid, and methyl 4,5-di-*O*-caffeoyl quinate) exhibited strong antioxidant capacity in the DPPH assay (10.4–18.2 µM) and displayed inhibitory activity against Cu^2+^-mediated LDL oxidation (1.8–2.3 µM), stronger than those obtained for the positive controls, BHT and caffeic acid [24]. In another study, 3,5-di-*O*-caffeoylquinic acid also demonstrated significant antioxidant properties in the DPPH assay, with an IC_50_ value of 3.63 µg/mL [74]. Two secoiridoid glucosides, eustomoruside and eustomoside, and one flavonoid, isoorientin, isolated from the whole plant *S. stellata* Cav. also displayed strong radical scavenging activities in DPPH with an IC_50_ value of 7.1–8.5 μg/mL compared to that for ascorbic acid (IC_50_ = 6.3 μg/mL) [75]. The polysaccharide fraction from the roots of *D. asperoides* also demonstrated antioxidant effects in DPPH and ABTS assays but with little potency; the EC_50_ value was 0.355 mg/mL in DPPH free radical scavenging activity and 5.867 mg/mL in ABT [14].

### 6.10. Anticancer Activity

The synthetic drugs used in chemotherapy not only have strong toxic effects on cancer cells, but they also have strong adverse side effects in chemotherapy. Many plant specialized metabolites are used in cancer therapies and new substances, and new plant species with potential anti-cancer activity are being sought.

Considerable attention has been noted regarding the cytotoxicity of *Dipsacus* and *Scabiosa* against various cancer cell lines including lung carcinoma A549, hepatoma Bel7402 and Hep3B, gastric carcinoma BGC-823, AGS, KATO III, MKN-45, and SNU-638, liver H157 and HepG2, colon cancer HCT-8, ovary cancer A2780, breast MCF-7, human breast cancer MCF-7 and MDB-MB-231, acute myeloid leukemia OCI-AML3, osteosarcoma HOS, or fibrosarcoma HT1080 cell lines. Some pure specialized metabolites of different classes identified in the plant extracts have also demonstrated potent or promising cytotoxic effects in vitro (Table 10) [11,12,40,48,56,75,81,89,90,96,173,174,175].

An aqueous extract of *D. asperoides* roots inhibited the viability of human mammary carcinoma-derived triple negative MDA-MB-231 cells (with IC_50_ = 15 μg/mL) and arrested the cell cycle in the G_2_/M phase; it also induced apoptosis by increasing pro-apoptotic caspase 3/7 activity and by suppressing the expression of BRAF, p-ERK, MEK, pPI3K, pAKT, and cyclin-dependent kinase 4/6 in a dose-dependent manner [175]. The cytotoxic activity of *bis*-iridoid glycosides fraction of *D. fullonum* leaf methanol extract (with sylvestroside III and IV as the main compounds) was evaluated against human breast cancer cell lines MCF7 and MDB-MB-231 and human cervical cancer HeLa cell line [174]. The two breast cancer cell lines were most sensitive to the fraction, resulting in a viability of 64.0% for MCF7 cells and 69.5% for MDB-MD-231 cells [174]. In addition, the ethanolic extracts of the aerial parts and flowers of *D. fullonum* have antiproliferative activity on the human hepatocellular carcinoma Hep3B cell line with an IC_50_ value above 100 µg/mL [176]. The methanol extract of *S. atropurpurea* subsp. *maritima* leaves at a concentration of IC_10_, IC_20_, or IC_30_ enhanced the toxicity of doxorubicin in human epithelial colorectal adenocarcinoma Caco-2 cells with IC_50_ = 1.04 µg/mL (vs. 2.41 µg/mL when the cells were treated only with doxorubicin) [81]. In addition, the combination of doxorubicin with *S. atropurpurea* extracts at a concentration of IC_50_ and IC_10_, respectively, increased the percentage of apoptotic cells, the percentage of caspase-activated cells, mRNA levels of the apoptosis related-genes (Bax, caspase-3, p21), and decreased the expression level of anti-apoptotic genes (Bcl-2). It was a stronger effect than that obtained for doxorubicin or *S. atropurpurea* extract alone. The plant methanol extract also reversed P-glycoprotein or multidrug resistance-associated protein in Caco-2 cells [81]. The use of silver nanoparticles with *S. atropurpurea* subsp. *maritima* water fruit extract was found to be promising anticancer agents with cytotoxic activity against the human multiple myeloma U266 cell line and the human breast cancer cell line MDA-MB-231. The silver nanoparticles inhibited the growth of cells in a concentration-dependent manner with IC_50_ values of 10 and 12 µg/mL, respectively [56].

Some specialized metabolites such as phenolic acids, triterpenoid derivatives, or iridoids isolated from *Dipsacus* or *Scabiosa* revealed cytotoxic effects in various cancer cell lines [11,12,40,48,75,89,90,96,173]. Phenolic acids, such as caffeic acid, 2,6-dihydroxycinnamic acid, vanillic acid, 2′-*O*-caffeoyl-d-glucopyranoside ester, and caffeoylquinic acid, demonstrated cytotoxic activity against five cancer cell lines (A549, Bel7402, BGC-823, HCT-8, and A2780) with IC_50_ values ranging from 3.883 µg/mL to 7.395 µg/mL. The positive control, fluorouracil (a known cytostatic compound), had an IC_50_ value of 0.177–0.695 µg/mL [89]. Akebia saponin PA from *D. asperoides* caused the death of various human gastric cancer cell lines (AGS, MKN-45, SNU-638, and KATO III) via both apoptosis and autophagy. The IC_50_ values were 24.1 µM (MKN-45 cells), 27.6 µM (SNU-638), 30.3 µM (AGS), and 36.5 µM (KATO III). In addition, akebia saponin PA increased the AGS cell number in the sub-G_1_ phase and activated caspase-3, cleavage of PARP-1, MAPK, and p38/c-Jun N-terminal kinase. Autophagy was induced through the PI3K/AKT/mTOR and AMPK/mTOR pathways [173]. Another saponin, akebia saponin D, was also found to induce cytotoxicity of the human monocyte-like histiocytic U937 cells in a concentration-dependent manner (0.1–1000 µM); it also enhanced the percentage of sub-G_1_ cells and increased Bax and p53 gene expression [90]. Saponin XII isolated from the roots of *D. japonicus* (1–2 µg/mL) suppressed the growth of acute myeloid leukemia OCI-AML3 cells; it stimulated apoptosis, increased the number of cells in the G_0_/G_1_ phase of the cell cycle, decreased the number of cells in the S and G_2_/M phases, and activated caspase-3 [40]. Some triterpenoid saponins and iridoids isolated from *S. stellata* whole plants were found to have cytotoxic effects against the fibrosarcoma HT1080 cell line [48,75]. Scabiostellatoside F, at a concentration of 12.0 mM, was able to inhibit HT1080 cell growth by 50% [48]. Other triterpenoid saponins, scabiostellatoside B, D, E, and H, were found to have an IC_50_ of 38–49 µM. In addition, scabiostellatoside A, C, and G were not cytotoxic at a concentration of 50 mM [48]. Yu et al. [12] found that some compounds isolated from *D. asper* roots such as ursane and oleanane type triterpenoids with a feruloyloxy group or an arabinosyl moiety at C-3 were more cytotoxic than arboinane-type triterpenoids against four tumor cell lines: lung A549, liver H157 and HepG2, and breast MCF-7. Moreover, the highest activity was shown by an ursane-type triterpenoid (3*β*-*O*-trans-feruloyl-2*α*-hydroxy-urs-12-en-28-oic acid) with IC_50_ values of 5.66 μM (H157), 9.36 μM (MCF-7), 9.5 μM (HepG2), and 12.8 μM (A549). The oleanane-type triterpenoid arabinoglycosides with a diacetylated sugar unit displayed cytotoxicity against A549 and H157 cell lines with IC_50_ values below 10 μM. The compounds with a free or monoacetylated sugar moiety demonstrated cytotoxic activity with IC_50_ values above 20 μM [12]. Another oleanane-type triterpenoid saponin isolated from *D. asper* roots (3-*O*-[*β*-d-xylopyranosyl-(1→4)-*β*-d-glucopyranosyl-(1→4)][α-l-rhamnopyranosyl-(1→3)]-*β*-d-glucopyranosyl-(1→3)-α-l-rhamnopyranosyl-(1→2)-α-l-arabinopyranosylhederagenin) displayed cytotoxicity against two lung cancer cells lines, A549 and H157, with IC_50_ values of 6.94 and 9.06 μM, respectively [96].

The 16 kDa water-soluble polysaccharide (ADAPW) isolated from *D. asperoides* roots had the ability to inhibit the growth of human osteosarcoma cell line HOS and induce apoptosis in a concentration-dependent manner (100, 200, and 400 µg/mL) after 24 h. It was also found to down-regulate PI3K and pAkt protein levels, reduce mitochondrial membrane potential, and increase intracellular ROS level [11].

However, a number of iridoid glycosides (dipsanosides C-G, 3′-*O*-*β*-d-glucopyranosyl sweroside, loganin, cantleyoside, triplostoside A, lisianthioside, and 6′-*O*-*β*-d-apiofuranosyl sweroside) had no cytotoxic effect on a set of tested cell lines, including lung carcinoma A549, hepatoma Bel7402, gastric carcinoma BGC-823, colon cancer HCT-8, and ovary cancer A2780 [89]. Similarly, 7-*O*-(*E*-*p*-coumaroyl)-sylvestroside I isolated from the whole plants of *S. stellata* also was not cytotoxic (IC_50_ > 100 μg/mL) to fibrosarcoma HT1080 cells. However, 7-*O*-(*E*-caffeoyl)-sylvestroside I showed moderate activity, with an IC_50_ value of 35.9 μg/mL [75].

Taken together, these above results indicated that some *Dipsacus* and *Scabiosa* plants or some specialized metabolites may display anticancer activity and may be useful as chemopreventive agents.

### 6.11. Antimicrobial and Anti-Insecticidal Activity

An increase in bacterial resistance to antibiotics has caused researchers to look for alternative solutions, which may be natural antibiotics [177].

Recent studies confirmed that extracts or essential oils from *Dipsacus* or *Scabiosa* spp. such as *D. asper*, *D. fullonum*, *D. japonicus*, *S. stellata*, *S. arenaria*, or *S. atropurpurea* subsp. *maritima* have antimicrobial activity [21,33,34,56,75,77,82,83,102]. Traditionally, *D. fullonum* is known as the remedy for Lyme disease caused by *Borrelia burgdorferi* whose vectors are ticks. The anti-*Borrelia* activity of *D. fullonum*/*D. sylvestris* extracts were evaluated in only a few studies in the recent ten years [34,178,179]. A 70% ethanol extract of *D. fullonum* leaves and its fractions showed significant anti-*Borrelia* activity against the stationary phase of *B. burgdorferi* strain B31 [34]. The strongest growth inhibition was found for a crude ethanol extract, which suppressed the cell viability by about 80% at a concentration of 305.5 mg/L. The NP5 fraction, containing loganic acid, and NP7, rich in saponarin, isoorientin, and two chlorogenic acid derivatives, were also effective, with a residual viability of 23.4–29.8% at a concentration of 332.8 mg/L and 340.2 mg/L, respectively; these values were comparable to that of the positive control, the triple antibiotic combination (doxycylin, cefoperazone, and daptomycin at a dose of 22.2 mg/L, 33.4 mg/L, and 80.1 mg/L, respectively) [34]. In contrast, Feng et al. [179] found that the 45% ethanolic extract of *D. fullonum* (accidentally mixed with a sample of *D. asper*) at a concentration of 0.25–1% was not active against either the non-growing stationary phase or growing *B. burgdorferi*, with residual viability of 84–90% and MIC > 2%. Among three tested extracts (70% ethanolic, ethyl acetate, and dichloromethane extracts) from *D. sylvestris* roots, only the ethanol extract was inactive against *B. burgdorferi* while the ethyl acetate extract showed the strongest ability [178]. A 50% methanolic extracts of *D. fullonum* leaves and roots were also tested against other microorganisms, including bacteria (*Bacillus subtilis* B5, *Escherichia coli* ATCC 10536, *Pseudomonas aeruginosa* DSM 939*, P. fluorescens* W1, and *Staphylococcus aureus* DSM 799) and yeasts (*Candida famata* AII4b, *C. tropicalis* ATCC 60557, *C. sphaerica* FII7A, *Saccharomyces cerevisiae* SV30, and *Yarrowia lipolytica* PII6a). It was found that the cell growth inhibitory activity differed among plant materials and bacteria strains. The greatest effect of growth inhibition zones was observed for the root extract against *E. coli* ATCC 10536 and *S. aureus* DSM 799 [33].

The antibacterial potential was also found for *S. arenaria* [82]. Various degree of antibacterial activity was related to the type of plant material (stems and leaves, roots, flowers, and fruits) and solvent used (crude extract and its fractions such as ethyl acetate, *n*-butanol, and aqueous). It was found that the highest antibacterial effect was noted for the *n*-butanolic fraction of fruits. In this case, MIC values for two *Escherichia coli* strains and two *Pseudomonas aeruginosa* strains were 0.019 mg/mL and 0.156 mg/mL, respectively. The butanolic fractions of the aerial parts and flowers were active against only *E. coli* strains with MIC values of 0.078 mg/mL and 0.156 mg/mL, respectively. In addition, *Staphylococcus aureus* ATCC 25923 and *S. saprophyticus* were sensitive to the butanolic fraction of fruits (MIC = 0.625 mg/mL). Among four tested strains of *Candida* spp. (*C. albicans* ATCC 90028, *C. glabrata* ATCC 90030, *C. parapsilosis* ATCC 22019, and *C. krusei* ATCC 6258), the most sensitive was *C. albicans* ATCC 90028 with MIC = 0.0195 mg/mL. *E. coli* ATCC 25922 and C. *albicans* ATCC 90028 were also sensitive to eleven subfractions from the butanolic fraction of the aerial part (MIC = 0.0195 mg/mL) [82]. A 70% ethanol extract of the whole plant *S. stellata* showed the highest antibacterial activity against *Streptococcus pyogenes* with MIC = 1.2 mg/mL (in comparison to gentamicin MIC = 2 µg/mL). For other strains of Gram-positive bacteria (*Bacillus subtilis*, *Enterococcus faecalis* ATCC 1034, *Staphylococcus aureus* 8325-4, *S. aureus* CIP 53.154, *S. epidermidis*, *Micrococcus luteus*, and *Listeria innocua*), Gram-negative bacteria (*Escherichia coli* CIP 54.127, *Enterobacter cloacae*, *Salmonella enterica*, *Serratia marcescens*, *Proteus vulgaris*, *Klebsiella pneumoniae*, *Providencia stuartii*, *Pseudomonas aeruginosa* ATCC 9027, and *Shigella sonnei*) and five yeasts (*Candida albicans*, *C. glabrata*, *C. tropicalis*, *C. kefyr*, and *Cryptococcus neoformans*), MIC ranged from 2.5 mg/mL to above 10 mg/mL [75]. The highest antimicrobial activity was found for fractions B and C obtained after eluting from a Diaion HP-20 column with 25% and 50% methanol. *Staphylococcus* spp., *Candida* spp. (*C. albicans*, *C. tropicalis*, and *C. kefyr*), and *Cryptococcus neoformans* were the most sensitive microorganisms to both fractions with MIC values of 0.6–1.5 mg/mL, while *E. faecalis* ATCC 1034*, M. luteus*, and *S. pyogenes* were also sensitive to fraction B [75]. The ethyl acetate, *n*-butanol, and the petroleum ether extracts from *S. stellata* whole plants were also tested for antibacterial activity in the agar disk diffusion assay against ten bacterial strains including four Gram-positive (*Staphylococcus aureus* ATCC 25923, *S. albus*, *Enterococcus* spp., and *Streptococcus* D) and six Gram-negative bacteria (*Escherichia coli* ATCC 35218*, Pseudomonas aeruginosa* ATCC 15442*, Acinetobacter baumannii, Proteus mirabilis, Salmonella typhimurium,* and *Enterobacter sakazaki*) [77]. Three bacterial strains, *S. albus*, *P. aregionosa* ATCC 15442, and *S. typhimurium*, were the most resistant strains to all extracts. The highest activity was exhibited by the ethyl acetate extract against the clinical strain of *P. mirabilis* (16–20 mm of the growth inhibition zones at a concentration of 0.0625–1 mg/mL). In addition, this extract also was active against five other bacterial strains, including *S. aureus* ATCC 25923, *A. baumannii*, *E. coli* ATCC 35218, *Enterococcus* sp., and *Streptococcus* D. The petroleum ether extract showed inhibitory activity against *S. aureus* (ATCC 25923) and *E. coli* (ATCC 35218) while the *n*-buthanol extract against *A. baumannii* and *E. sakazaki* [77]. In another study, the antibacterial and antifungal activities of the silver nanoparticles with *S. atropurpurea* subsp. *maritima* water extract from fruit against bacteria (*Escherichia coli*, *Micrococcus luteus*, *Staphylococcus aureus*, and *Klebsiella pneumoniae*) and fungal pathogens including *Candida* clinical strains (*C. albicans*, *C. tropicalis*, and *C. glabrata*), *Microsporum canis*, *Trichophytom rubrum,* and *Trichophytom interdigitale* were also reported. The silver nanoparticles inhibited the cell growth of bacteria and *Candida* sp., as evidenced by the zone inhibition (19.3–28 mm) and the MIC value (3.9–15.62 µg/mL). The lowest MIC value was found for two dermatophyte species, *T. rubrum* and *T. interdigitale*. In addition, the antifungal potential of the silver nanoparticles was associated with the disruption of membrane integrity and attenuation of the biofilm and hyphae formation [56]. *D. asper* crude extract from the roots also displayed antifungal activity in vivo in a whole-plant assay. This property was evaluated against seven plant pathogenic fungi such as *Magnaporthe grisea* causing rice blast, *Rhizoctonia solani* causing rice sheath blight, *Botrytis cinerea* causing tomato gray mold, *Phytophthora infestans* causing tomato late blight, *Puccinia recondita* causing wheat leaf rust, *Blumeria graminis* f. sp. *hordei* causing barley powdery mildew, and *Colletotrichum coccodes* causing red pepper anthracnose. It was shown that the activity was dependent on the fungal pathogens and the solvent used (*n*-hexane, ethyl acetate, acetone, methylene chloride, and methanol) for extraction. The fungi causing the tomato late blight and the tomato gray mold were the most sensitive to *Dipsacus* root extract. The greatest anti-fungal effect was demonstrated by the ethyl acetate and acetone extracts at a concentration of 1–2 mg/mL that inhibited tomato diseases by 90% [21].

Antifungal activity was also demonstrated by the pure compounds isolated from the roots of *D. asper* such as cauloside A (the main compound of the extract). Cauloside A was most effective against fungal pathogens causing the tomato late blight, the rice blast, and the tomato gray mold at a dose of 0.5 mg/mL. Colchiside inhibited the growth of *Phytophthora infestans* while three sterols (campesterol, β-sitosterol, and stigmasterol) displayed the weakest antifungal activity [21]. Among twelve specialized metabolites isolated from the whole plant *S. stellata*, two iridoids, viz., 7-*O*-caffeoyl-sylvestroside I and 7*-O*-(*p*-coumaroyl)-sylvestroside I, showed the highest antimicrobial activity with an MIC value of 31.2 µg/mL against *Enterococcus faecalis* ATCC 1054 and *Staphyllococcus epidermis*; sylvestroside I was also able to inhibit the growth of *E. coli* CIP 54.127 (MIC = 62.5 µg/mL). These iridoids also inhibited the growth of *S. aureus* CIP 53.154 with an MIC value of 62.5 µg/mL [75]. 2′,4′-*O*-diacetyl-3-*O*-*α*-l-arabinopyranosyl-23-hydroxyolea-12-en-28-oic acid and hederagonic acid, isolated from *D. asper* roots, inhibited the growth of *S. aureus* ATCC 25923 with IC_50_ values of 12.3 and 10.3 µM, respectively. Furthermore, 2*α*,3*β*,24-trihydroxy-23-norurs-12-en-28-oic acid and 2*α*,3*β*-dihydroxy-23-norurs-4(24),11,13(18)-trien-28-oic acid also exhibited antimicrobial activity but the IC_50_ value was three-times higher [12]. The other triterpenoid derivative, oleanolic acid, found in some *Dipsacus* and *Scabiosa* species, showed weaker antibacterial properties against *E. coli* ATCC 25922, *Pseudomonas aeruginosa* ATCC 27853, and *Candida albicans* ATCC 90028 with an IC_50_ value ranging from 170 µM to 680 µM [82].

It is well known that essential oils and their ingredients have potent antimicrobial properties [177]. The essential oil isolated from flowers of *S. arenaria* showed a strong ability (stronger than the positive control, thymol; MIC = 0.2 mg/mL) to inhibit the growth of cells of two *Staphylococcus aureus* strains with an MIC = 0.1562 mg/mL [83]. Notably, the essential oil isolated from fruits was found to be an anticandidal agent against *Candida albicans* ATCC 90028, *C. parapsilosis* ATCC 27853, *C. kreusei* ATCC 6258, and *C. glabrata* ATCC 90030 (MIC = 0.625 mg/mL) [83]. The essential oil isolated from *D. japonicus* flowering aerial parts can be used as a promising, natural insecticidal agent against stored-product insects such as adult red flour beetles (*Tribolium castaneum*) and maize weevils (*Sitophilus zeamais*), and they displayed contact toxicity with LD_50_ values of 13.45 μg/adult and 18.32 μg/adult, respectively. This essential oil also had fumigant activity against adult insects with LC_50_ 5.26 mg/l air for *T. castaneum* and 10.11 mg/l air for *S. zeamais*. The strongest fumigant toxicity was possessed by one of the abundant ingredients in *D. japonicus* essential oil, i.e., 1,8-cineole [102].

Regarding antiviral activity, only one study showed that dipsalignan A, (+)-1-hydroxy-2,6-bis-*epi*-pinoresinol, and dipsanosides M-N displayed inhibitory activities against human immunodeficiency virus-1 (HIV-1) integrase. The IC_50_ values were 53.26 μM, 61.74 μM, 84.03 μM, and 92.67 μM, respectively. The positive control, baicalein, had a value of 1.37 μM [13].

### 6.12. Others

Akebia saponin D was also found to be a potential antidepressant agent. Intraperitoneal injection (40 mg/kg/d) alleviated LPS-induced microglia-mediated neuroinflammatory response in mice by inhibiting the TLR4/NF-κB signaling pathway in the hippocampus and prefrontal cortex [180]. It also ameliorated chronic mild stress-induced depressive-like behaviors in C57BL/6 mice by inducing a neuroprotective microglial phenotype in the hippocampus through the PPAR-γ pathway [181]. A similar antidepressant effect was also found for apigenin in a mouse model of chronic mild stress [182].

Gong et al. [32] found akebia saponin D to be effective against pain. It displayed an anti-nociceptive effect in SPF KM mice by shortening the licking time in the formalin test, increasing the reaction time to heat stimuli, and inhibiting acetic acid-induced writhing in mice.

Akebia saponin D activated the expression of the progesterone receptor in primary decidual cells and the Notch signaling pathway. Gao et al. [62] proposed that *Dipsaci radix* and its main ingredient, akebia saponin D, may promote decidualization in pregnant women. Bushen Antai, a Chinese herbal medicine preparation containing *Dipsaci radix*, was found to reduce the pregnancy loss caused by mifepristone administration [183]. This preparation may stimulate estrogen and progesterone receptors through Akt and Erk1/2 signaling pathways in the maternal–fetal interface of pregnant rats.

## 7. Conclusions

The present review broadens the knowledge of the phytochemistry of some species of *Dipsacus* and *Scabiosa* genera as well as their biological properties. The phytochemical analyses showed qualitative similarities in some specialized metabolites, especially iridoids, between species of both genera. Some species of *Dipsacus* and *Scabiosa* contain above 200 different compounds belonging to iridoids, triterpenoids derivatives, flavonoids, or phenolic acids with caffeoylquinic acid derivatives. *Dipsacus* spp. were predominated by terpenoid saponins while *Scabiosa* spp. were rich sources of iridoids and flavonoids. Apigenin, luteolin, and their derivatives were particularly common. Hederagenin and its related saponins are the main group of triterpenoids identified in *Dipsacus*. The oleanane-type triterpenoids were also common in *Scabiosa* genus. The wine-processing method has a beneficial effect on the biological activity of *Dipsaci radix* and the level of some specialized metabolites with its quality indicator, akebia saponin D. *Dipsacus,* and *Scabiosa* species and their constituent compounds possess beneficial biological activities. Many in vitro and in vivo studies confirmed their traditional medicinal uses. *Dipsaci radix* and akebia saponin D demonstrated anti-osteoporosis and antiarthritic properties. *Scabiosa* spp. showed anti-hepatic fibrosis potential. In addition, akebia saponin D displayed cardioprotective activity. Antioxidant, antimicrobial, and anti-inflammatory activities of both genera were also confirmed. Some newly identified specialized metabolites such as polysaccharides displayed promising biological properties. Thus, in the future, it is worth paying more attention to their pharmacological activities. The varied biological activities of extracts from *Dipsacus* and *Scabiosa* as well as the pure compounds isolated from them indicate their potential use in the future as effective, natural herbal drugs in the treatment of various diseases in official medical applications.

## Figures and Tables

**Table 1:** The common names, natural occurrence, and traditional usage of *Dipsacus* and *Scabiosa* species.

Accepted Species Name (Synonym Name ^a^)	Common Name	Geographical Occurrence	Habitat	Traditional Usage (Part of Plant)
*D. asperoides* C.Y.Cheng & T.M.Ai [8] *D. asper* Wall. ex DC. [9] *D. asper* Wall. [4] (*D. asper* Wall. ex C.B.Clarke (doubtful) [4])	Roots are called Xu duan, chuan xu duan, (in Chinese); Sichuan teasel, Himalayan Teasel Root (in English) [4,10,11,12,13,14,15,16,17]	China (Hubei, Hunan, Yunnan, Shanxi, Jiangxi, Sichuan, Gansu, Xizang, Guangxi, and Guizhou Provinces), Korea, Japan, Myanmar [9,12,18,19,20,21,22,23]	Moist fields, margins of forests, thickets, among herbs, by streams, roadsides, and mountains; 1500–3700 m [10,24,25]	Roots are used in traditional Chinese and Korean medicine to treat low back pain, knee pain, osteoporosis or bone diseases, bone fractures, rheumatic arthritis, lumbago, traumatic hematoma, uterine bleeding, and threatened abortion [6,11,14,15,22,26,27,28,29,30,31,32]
*D. fullonum* L. [4,8,9] (*D. sylvestris* Huds. [8,9])	Cardo, Fuller’s teasel, teasel, wild teasel (in English) [4,9,33,34]	Europe to the Caucasus, North-Western Africa [9]	-	Herb is used for treatment of Lyme disease [35] and for eye infection in cattle (in Sardinia) [36]
*D. inermis* Wall. [4,8,9] (*D. asper* Wall. ex. DC. [8])	zang xu duan (in Chinese); Wopal haakh/Wopal Hak (in Kashmiri) [10,37,38]	Afghanistan, Bangladesh, India, People’s Republic of China (Yunnan Province), Myanmar, Nepal, Pakistan, Thailand, Vietnam, Kashmir Himalaya [9,10,37]	Forests, grassy slopes, by streams; 2100–3900 m [10]	Himalayan herb is used in traditional Ayurvedic medicine and Kashmiri traditional medicine against various inflammation-related disorders, body weakness, cough, and sore throat. It has stomachic and carminative properties [37,38,39]
*D. japonicus* Miq. [4,8,9]	ri ben xu duan, (in Chinese); Japanese teasel (in English) [4,10]	People’s Republic of China, Korea, central and south Japan, and Vietnam [9,40]	Grassy slopes and roadsides, savannas; below 1000–2600 m [10,41]	Roots are used in the traditional medicine of China and Vietnam as a remedy for relieving joint pain and inflammation [40]
*D. sativus* (L.) Honc. [4,8,9]	Fuller’s Teasel (in English) [4]	Europa (France, Italy), Caucasus [9]	-	Tea of leaves is used for the treatment of cardiovascular diseases [42]
*S. arenaria* Forssk. [8,9]	-	Algeria, Egypt, Libya, Morocco, Palestine, Sinai, and Tunisia [9]	-	-
*S. atropurpurea* L. [8,9]	Mourningbride (in English); Mor uyuzotu or Şeytanotu (in Turkey); Ambarina (in Northern Peru); Escabiosa (in north-east Catalonia, Iberian Peninsula) [4,43,44,45]	Algeria, Tunisia, Turkey, Europe (Albania, Azores, Baleares, Bulgaria, Canary Is., Corse, East Aegean Is., France, Greece, Italy, Madeira, Morocco, Portugal, Sicilia, Spain) [9]	Around roadsides, dry fields, and dunes [45]	Flowers/aerial parts are used traditionally in Catalonia for bronchitis, acne, cold and cough, and for measles and furuncleas, in Northern Peru for menstrual regulation, and in the Iberian Peninsula as a veterinary diuretic [43,44,45,46]
*S. comosa* Fisch. ex Roem & Schult. [8,9]	lan pen hua, (in Chinese) [10]	People’s Republic of China (Gansu, Hebei, Henan, Heilongjiang, Jilin, Liaoning, Shaanxi, Shanxi Provinces, Nei, and Ningxia), Korea, Inner Mongolia, Russia (Far East, Siberia) [9,10]	Sandy dunes, dry mountain slopes, steppes; 300–1600(–3000) m [10]	In traditional Mongolian and Tibetan medicine, inflorescences are used in the treatment of liver diseases [7,47]
*Lomelosia stellata* (L.) Raf. [4,8,9] (*S. stellata* L. [8,9])	Starflower pincushions (in English) [4,48]	Endemic to North Africa (Algeria, Libya, Morocco, Tunisia), Europe (France, Portugal, Spain) [9,48]	Dry sunny grassland and rocky hillsides [49]	Leaves and flowers are used in the traditional medicine of Morocco to treat heel cracks and for the treatment of various respiratory diseases including bronchitis, bronchial pneumonia, influenza, and asthma [48,50]
*S. tschiliensis* Grüning [8,9] (synonymous name of *S. comosa* Fisch. ex Roem & Schult. [8,9])	Meng Gu Shan Luo Bo (in Chinese) [51]	Inner Mongolia autonomy district and China (the west of Hebei Province) [52]	Mountainous regions (300–1500 m) [52,53]	Inflorescences are used in inner Mongolia for the treatment of headache, fever, cough, and jaundice [51]

^a^ names of species cited by the authors of publications used in this review, which are synonymous names according to The World Flora Online [8], Plants of the World Online [9], or The Global Biodiversity Information Facility [4].

**Table 9:** Antioxidant potential of some *Dipsacus* and *Scabiosa* species.

Species	Plant Material	Extract, Fraction, or Pure Compound	Antioxidant Assay	Antioxidant Activity	Positive Control	References
*D. asper*	Roots	Methanolic extract; 3,4-di-*O*-caffeoylquinic acid; methyl 3,4-di-*O*-caffeoyl quinate; 3,5-di-*O*-caffeoylquinic acid; methyl 3,5-di-*O*-caffeoyl quinate; 4,5-di-*O*-caffeoylquinic acid; methyl 4,5-di-*O*-caffeoyl quinate	DPPH; Cu^2+^ mediated low-density lipoprotein (LDL)	DPPH: methanolic extract IC_50_ = 90.2 µg/mL; 3,4-di-*O*-caffeoylquinic acid IC_50_ = 13.4 µM; methyl 3,4-di-*O*-caffeoyl quinate IC_50_ = 14.1 µM; 3,5-di-*O*-caffeoylquinic acid IC_50_ = 18.2 µM; methyl 3,5-di-*O*-caffeoyl quinate IC_50_ = 10.6 µM; 4,5-di-*O*-caffeoylquinic acid IC_50_ = 10.4 µM; methyl 4,5-di-*O*-caffeoyl quinate IC_50_ = 12.6 µM Cu^2+^ mediated LDL: methanolic extract IC_50_ = 134.4 µg/mL; 3,4-di-*O*-caffeoylquinic acid IC_50_ = 2.1 µM; methyl 3,4-di-*O*-caffeoyl quinate IC_50_ = 1.9 µM; 3,5-di-*O*-caffeoylquinic acid IC_50_ = 2.3 µM; methyl 3,5-di-*O*-caffeoyl quinate IC_50_ = 2.0 µM; 4,5-di-*O*-caffeoylquinic acid IC_50_ = 2.3 µM; methyl 4,5-di-*O*-caffeoyl quinate IC_50_ = 1.8 µM	DPPH: BHT IC_50_ = 145.8 µM; caffeic acid IC_50_ = 31.1 µM Cu^2+^ mediated LDL: BHT IC_50_ = 3 µM; caffeic acid IC_50_ = 5.2 µM; α-tocopherol IC_50_ = 23.4 µM	[24]
*D. asperoides*	Roots	Polysaccharide fraction	DPPH; ABTS	DPPH: EC_50_ = 0.355 mg/mL (at concentration 1 mg/mL 86.2% scavenging rate) ABTS: EC_50_ = 5.867 mg/mL (at concentration 10 mg/mL 92.6% scavenging rate)	-	[14]
*D. fullonum*	Whole plants	Acetone:water extract (7:3)	DPPH; ABTS	DPPH: 4.01 mmol TEAC/100 g d.w. ABTS: 3.58 mmol TEAC/100 g d.w.	-	[171]
	Leaves	Aqueous extract; aqua [TEAH]^+^[Thr]^−^; aqua [TEAH]^+^[Met]^−^	DPPH; FRAP; CUPRAC	DPPH: aqua radical scavenging activity (RSA) 68.11%–73.81%; aqua [TEAH]^+^[Thr]^−^ 57.55%–62.78%; aqua [TEAH]^+^[Met]^−^ 58.73%–61.17% FRAP: aqua 13.14–14.85 mg FeSO_4_/g raw material; aqua [TEAH]^+^[Thr]^−^ 1.45–17.08 mg FeSO_4_/g raw material; aqua [TEAH]^+^[Met]^−^ 12.44–21.1 mg FeSO_4_/g raw material; CUPRAC: aqua 4.93–5.71 mg TEAC/g raw material; aqua [TEAH]^+^[Thr]^−^ 5.08–5.74 mg TEAC/g raw material; aqua [TEAH]^+^[Met]^−^ 4.39–6.09 mg TEAC/g raw material	-	[172]
	Leaves	70% ethanol extract (*v*/*v* %); fraction NP7 (contained two chlorogenic acid derivatives, saponarin and isoorientin); fraction NP2 (contained *bis*-iridoids)	ORAC	70% ethanol extract 10.8 mmol TEAC/100 mL; NP7 fraction 12.5 mmol TEAC/100 mL; NP2 fraction 0.78 mmol TEAC/100 mL	-	[34]
	Leaves	50% methanol extract	ORAC	14.78 mmol TEAC/100 g dry weight	-	[33]
	Roots	50% methanol extract	ORAC	10.87 mmol TEAC/100 g dry weight	-	[33]
*D. sativus*	Leaves	Methanol extract	In vivo study with ICR mice pre-treated with D-galactose	SOD level in the peripheral blood plasma ↑; MDA level in the peripheral blood plasma ↓	-	[42]
*S. arenaria*	Roots	80% (*v*/*v*) hydro-methanolic extract; *n*-butanol, ethyl acetate, and water fractions	DPPH; ABTS; reducing power; β-carotene bleaching inhibition	DPPH: 80% methanolic extract IC_50_ = 260 µg/mL; *n*-butanol fraction IC_50_ = 26 µg/mL; ethyl acetate fraction IC_50_ = 19 µg/mL; water fraction IC_50_ = 105 µg/mL ABTS: 80% methanolic extract IC_50_ = 210 µg/mL; *n*-butanol fraction IC_50_ = 40 µg/mL; ethyl acetate fraction IC_50_ = 34 µg/mL; water fraction IC_50_ = 180 µg/mL reducing power: 80% methanolic extract EC_50_ = 190 µg/mL; *n*-butanol fraction EC_50_ = 28 µg/mL; ethyl acetate fraction EC_50_ = 66 µg/mL; water fraction IC_50_ = 510 µg/mL β-carotene bleaching inhibition: 80% methanolic extract IC_50_ = 720 µg/mL; *n*-butanol fraction IC_50_ = 18 µg/mL; ethyl acetate fraction IC_50_ = 26 µg/mL; water fraction IC_50_ = 450 µg/mL	DPPH: BHT IC_50_ = 18 µg/mL; ABTS: BHT IC_50_ = 50 µg/mL; reducing power: BHT EC_50_ = 20 µg/mL; β-carotene bleaching inhibition: BHT IC_50_ = 40 µg/mL	[73]
	Stem and leaves; flowers; fruits	80% (*v*/*v*) hydro-methanolic extract; *n*-butanolic, ethyl acetate, and water fractions	DPPH; ABTS; reducing power; β-carotene bleaching inhibition	DPPH: steam and leaves 80% methanolic extract IC_50_ = 21 µg/mL; *n*-butanol fraction IC_50_ = 19 µg/mL; ethyl acetate fraction IC_50_ = 19 µg/mL; water fraction IC_50_ = 48 µg/mL; DPPH: flowers 80% methanolic extract IC_50_ = 36 µg/mL; *n*-butanol fraction IC_50_ = 19 µg/mL; ethyl acetate fraction IC_50_ = 17 µg/mL; water fraction IC_50_ = 30 µg/mL; DPPH: fruits 80% methanolic extract IC_50_ = 32 µg/mL; *n*-butanol fraction IC_50_ = 20 µg/mL; ethyl acetate fraction IC_50_ = 18 µg/mL; water fraction IC_50_ = 25 µg/mL; ABTS: steam and leaves 80% methanolic extract IC_50_ = 810 µg/mL; ethyl acetate fraction IC_50_ = 110 µg/mL; ABTS: flowers *n*-butanol fraction IC_50_ = 140 µg/mL; ethyl acetate fraction IC_50_ = 54 µg/mL; ABTS: fruits ethyl acetate fraction IC_50_ = 170 µg/mL; β-carotene bleaching inhibition: steam and leaves 80% methanolic extract IC_50_ = 920 µg/mL; *n*-butanol fraction IC_50_ = 400 µg/mL; ethyl acetate fraction IC_50_ = 210 µg/mL; β-carotene bleaching inhibition: flowers 80% methanolic extract IC_50_ = 900 µg/mL; *n*-butanol fraction IC_50_ = 180 µg/mL; ethyl acetate fraction IC_50_ = 52 µg/mL; β-carotene bleaching inhibition: fruits 80% methanolic extract IC_50_ = 870 µg/mL; *n*-butanol fraction IC_50_ = 700 µg/mL; ethyl acetate fraction IC_50_ = 680 µg/mL; water fraction IC_50_ = 800 µg/mL; reducing power: steam and leaves 80% methanolic extract EC_50_ = 240 µg/mL; *n*-butanol fraction EC_50_ = 220 µg/mL; ethyl acetate fraction EC_50_ = 100 µg/mL; water fraction EC_50_ = 220 µg/mL; reducing power: flowers 80% methanolic extract EC_50_ = 40 µg/mL; *n*-butanol fraction EC_50_ = 26 µg/mL; ethyl acetate fraction EC_50_ = 20 µg/mL; water fraction EC_50_ = 77 µg/mL; reducing power: fruits *n*-butanol fraction EC_50_ = 800 µg/mL; ethyl acetate fraction EC_50_ = 52 µg/mL; water fraction EC_50_ = 64 µg/mL	DPPH: BHT IC_50_ = 18 µg/mL; ABTS: BHT EC_50_ = 50 µg/mL; β-carotene bleaching inhibition: BHT IC_50_ = 40 µg/mL; reducing power: BHT EC_50_ = 20 µg/mL	[72]
*S. artropurpurea*	Stems	Dichloromethane, chloroform, ethyl acetate, ethanol extracts; volatile fractions/hydro distillation V1 hexane, V2 chloroform	DPPH	Dichloromethane extract IC_50_ = 2.7085 mg/mL; chloroform extract IC_50_ = 2.0951 mg/mL; ethyl acetate extract IC_50_ = 0.4806 mg/mL; ethanol extract IC_50_ = 0.1383 mg/mL; V1 fraction IC_50_ = 0.4798 mg/mL; V2 fraction IC_50_ = 1.2944 mg/mL	Ascorbic acid IC_50_ = 0.084 mg/mL	[46]
*S. artropurpurea* subsp. *maritima*	Fruits	Water extract (silver nanoparticles)	DPPH; FRAP	DPPH: IC_50_ = 0.112 mg/mL FRAP; IC_50_ = 0.036 mg EAa/g d.w.	DPPH: ascorbic acid IC_50_ = 0.087 mg/mL; FRAP: ascorbic acid IC_50_ = 0.024 mg EAa/g d.w.	[56]
*S. comosa*	Inflorescences	70% ethanol extract; 3,5-di-*O*-caffeoylquinic acid; chlorogenic acid; 4,5-di-*O*-caffeoylquinic acid; 3,4-di-*O*-caffeoylquinic acid; caffeic acid; luteolin-7-glucoside; luteolin-6-C-glucoside; quercetin-3-glucoside; quercetin-3-rutinoside	DPPH; ABTS	DPPH: 70% ethanol extract IC_50_ = 331.1 µg/mL; 3,5-di-*O*-caffeoylquinic acid IC_50_ = 3.63 µg/mL; chlorogenic acid IC_50_ = 4.67 µg/mL; 4,5-di-*O*-caffeoylquinic acid IC_50_ = 4.01 µg/mL; 3,4-di-*O*-caffeoylquinic acid IC_50_ = 4.78 µg/mL; caffeic acid IC_50_ = 3.96 µg/mL; protocatechuic acid IC_50_ = 4.15 µg/mL; luteolin IC_50_ = 6.03 µg/mL; luteolin-7-glucoside IC_50_ = 9.16 µg/mL; luteolin-6-C-glucoside IC_50_ = 5.74 µg/mL; quercetin-3-glucoside IC_50_ = 6.47 µg/mL; quercetin-3-rutinoside IC_50_ = 6.15 µg/mL ABTS: 70% ethanol extract IC_50_ = 223.5 µg/mL	-	[74]
*S. stellata*	Whole plants	70% ethanol extract; fraction A 100% water; fraction B 25% methanol; fraction C 50% methanol; fraction D 75% methanol; fraction E 100% methanol; eustomoruside; eustomoside; hyperin	DPPH	70% ethanol extract IC_50_ = 86 µg/mL; fraction A IC_50_ = 133 µg/mL; fraction B IC_50_ = 48.7 µg/mL; fraction C IC_50_ = 25 µg/mL; fraction D IC_50_ = 64.3 µg/mL; fraction E IC_50_ >200 µg/mL; eustomoruside IC_50_ = 7.1 µg/mL; eustomoside IC_50_ = 7.2 µg/mL; hyperin IC_50_ = 16 µg/mL	Ascorbic acid IC_50_ = 6.3 µg/mL	[75]
	Whole plants	Petroleum ether; ethyl acetate; *n*-butanol extract	DPPH; ABTS; FRAP; CUPRAC; β-carotene; phosphomolybdate; ferrous and metal ions chelating	DPPH: petroleum ether IC_50_ = 171.61 µg/mL; ethyl acetate IC_50_ = 25.15 µg/mL; *n*-butanol extract IC_50_ = 21.22 µg/mL ABTS: petroleum ether IC_50_ = 64.1 µg/mL; ethyl acetate IC_50_ = 14.00 µg/mL; *n*-butanol extract IC_50_ = 24.99 µg/mL CUPRAC: petroleum ether IC_50_ = 100.95 µg/mL; ethyl acetate IC_50_ = 28.5 µg/mL; *n*-butanol extract IC_50_ = 42.16 µg/mL β-carotene: petroleum ether IC_50_ = 11.18 µg/mL; ethyl acetate IC_50_ = 10.34 µg/mL n; *n*-butanol extract IC_50_ = 50.01 µg/mL chelation in ferrous iron: ethyl acetate EC_50_ = 5.026 mg/mL n; *n*-butanol extract EC_50_ = 1.652 mg/mL chelation in metal iron: ethyl acetate EC_50_ > 200 mg/mL n; *n*-butanol extract EC_50_ = 145.35 mg/mL	DPPH: BHA IC_50_ = 6.82 µg/mL; BHT IC_50_ = 22.32 µg/mL; tannic acid IC_50_ = 7.74 µg/mL; ascorbic acid IC_50_ = 3.1 µg/mL; α-tocopherol IC_50_ = 13.02 µg/mL ABTS: BHA IC_50_ = 1.81 µg/mL; BHT IC_50_ = 1.29 µg/mL; tannic acid IC_50_ = 1.01 µg/mL; ascorbic acid IC_50_ = 1.74 µg/mL; α-tocopherol IC_50_ = 7.59 µg/mL CUPRAC: BHA IC_50_ = 3.64 µg/mL; BHT IC_50_ = 9.62 µg/mL; tannic acid IC_50_ = 3.76 µg/mL; ascorbic acid IC_50_ = 12.43 µg/mL; α-tocopherol IC_50_ = 19.92 µg/mL chelation in ferrous iron: EDTA EC_50_ = 0.0627 µg/mL chelation in metal iron: EDTA EC_50_ = 8.57 mg/mL	[50,77]
	Whole plants	*n*-butanol, ethyl acetate, dichloromethane fractions	DPPH; ABTS; FRAP	DPPH: *n*-butanol FRS_50_ = 64.46µg/mL; ethyl acetate FRS_50_ = 71.82 µg/mL; dichloromethane FRS_50_ > 250 µg/mL ABTS: *n*-butanol FRS_50_ = 27.87 µg/mL; ethyl acetate FRS_50_ = 40.41 µg/mL; dichloromethane FRS_50_ > 250 µg/mL FRAP: *n*-butanol EC_50_ = 161.11 µg/mL; ethyl acetate EC_50_ = 202.41 µg/mL; dichloromethane EC_50_ > 50 µg/mL	DPPH: ascorbic acid FRS_50_ = 8.21 µg/mL ABTS: Trolox FRS_50_ = 12.07 µg/mL FRAP: BHA EC_50_ = 18.03 µg/mL	[76]
*S. tschilliensis*	Inflorescences	70% ethanol extract	DPPH; ABTS	DPPH: IC_50_ = 272.8 µg/mL ABTS: IC_50_ = 199.7 µg/mL	-	[74]
	Whole plants	90% ethanol extract	DPPH	DPPH: IC_50_ = 26.502 µg/mL	Ascorbic acid IC_50_ = 5.41 µg/mL	[52]
	Whole plants in pre-flowering, flowering, and fruiting stages	96% ethanol extract; water, *n*-butanol, ethyl acetate, petroleum ether fractions	DPPH; ABTS; inhibition of lipid peroxidation; OH scavenging activity	DPPH: 96% ethanol extract IC_50_ = 25.65–86.79 µg/mL; acetate fraction of pre-flowering stage of plants IC_50_ = 8.47 µg/mL ABTS: acetate fraction of pre-flowering stage of plants IC_50_ = 58.76 µg/mL OH scavenging activity: 96% ethanol extract IC_50_ = 206.47–772.45 µg/mL; acetate fraction of pre-flowering stage of plants IC_50_ = 67.64 µg/mL	DPPH: ascorbic acid IC_50_ = 7.6 µg/mL; ABTS: lipid peroxidation inhibition: OH scavenging activity:	[53]

ABTS, 2,2′-azinobis(3-ethylbenzothiazoline-6-sulfonic acid) radical cation assay; BHA, butylated hydroxyanisole; BHT, butylhydroxytoluene; CUPRAC, cupric-reducing antioxidant capacity; DPPH, 2,2-Diphenyl-1-picrylhydrazyl radical scavenging assay; EAa, equivalent ascorbic acid; EC_50_ and IC_50_, the half maximal effective and inhibitory concentration of sample, respectively; FRAP, ferric reducing antioxidant assay; FR, free radical scavenge; LDL, low-density lipoprotein; ORAC, oxygen radical absorbance capacity; TEAC, Trolox equivalent antioxidant capacity; ↓, decrease; ↑, increase.

**Table 10:** Anticancer activities of some species of *Dipsacus* and *Scabiosa*.

Species	Plant Material	Extract or Specialized Metabolites	Cell Line	Assay	Result	Positive Control	References
*D. asper*	Roots	Phenolic acids (2,6-dihydroxycinnamic acid, vanillic acid, 2′-*O*-caffeoyl-d-glucopyranoside ester, caffeoylquinic acid) iridoids (dipsanosides C-G, 3′-O-β-d-glucopyranosyl sweroside, loganin, cantleyoside, triplostoside A, lisianthioside, and 6′-*O*-β-d-apiofuranosyl sweroside)	Lung carcinoma A549, hepatoma Bel7402, gastric carcinoma BGC-823, colon cancer HCT-8, and ovary cancer A2780	MTT assay, cell viability (96 h)	2,6-dihydroxycinnamic acid, A549 IC_50_ = 3.883 µg/mL, Bel7402 IC_50_ = 7.346 µg/mL, BGC-823 IC_50_ = 4.321 µg/mL; vanillic acid, Bel7402 IC_50_ = 6.437 µg/mL, HCT-8 IC_50_ = 5.218 µg/mL, A2780 IC_50_ = 7.395 µg/mL; 2′-*O*-caffeoyl-d-glucopyranoside ester, A549 IC_50_ = 5.663 µg/mL; Bel7402 IC_50_ = 5.545 µg/mL, BGC-823 IC_50_ = 6.432 µg/mL, HCT-8 IC_50_ = 5.7 µg/mL, A2780 IC_50_ = 6.380 µg/mL; caffeoylquinic acid, A549 IC_50_ = 5.713 µg/mL; Bel7402 IC_50_ = 5.586 µg/mL, BGC-823 IC_50_ = 6.204 µg/mL, HCT-8 IC_50_ = 5.37 µg/mL, A2780 IC_50_ = 6.679 µg/mL iridoids were not cytotoxic against A549, Bel7402, BGC-823, HCT-8, and A2780 cell lines	Fluorouracil, A549 IC_50_ = 0.177 µg/mL, Bel7402 IC_50_ = 0.542 µg/mL, BGC-823 IC_50_ = 0.695 µg/mL, HCT-8 IC_50_ = 0.67 µg/mL, A2780 IC_50_ = 0.569 µg/mL	[89]
	Roots	Akebia saponin D	Human monocyte-like histiocytic U937 cells	MTT assay, cytotoxicity (48 h); flow cytometric analysis and DNA ladding (level of DNA fragmentation, sub-G1 peak); RT-PCR	Induction of cell cytotoxicity in a concentration-dependent manner (0.1–1000 µM); an increase in the percentage of Sub G_1_ cells and Bax and p53 gene expression	Doxorubicin, the cell viability 25% at a concentration of 0.1 µM	[90]
	Roots	2′,3′-*O*-diacetyl-3-*O*-α-l-arabinopyranosyl-23-hydroxyolea-12-en- 28-oic acid; 2′,4′-*O*-diacetyl-3-*O*-α-l-arabinopyranosyl-23-hydroxyolea-12-en- 28-oic acid; 3*β*-*O*-trans-feruloyl-2*α*-hydroxy-urs-12-en-28-oic acid; leontoside A; 4′-*O*-acetyl-3-*O*-*α*-l-arabinopyranosyl-23-hydroxyolea-12-en-28-oic acid; 3′-*O*-acetyl-3-*O*-*α*-l-arabinopyranosyl-23-hydroxyolea-12-en-28-oic acid; 2′-*O*-acetyl-3-*O*-*α*-l-arabinopyranosyl-23-hydroxyolea-12-en-28-oic acid	Lung A549, liver H157 and HepG2, and breast MCF-7 cell lines	SRB method, cytotoxicity (48 h)	2′,3′-*O*-diacetyl-3-*O*-α-L-arabinopyranosyl-23-hydroxyolea-12-en-28-oic acid, A549 IC_50_ = 6.67 µM; H157 IC_50_ = 9.7 µM; HepG2 IC_50_ = 15.89 µM; MCF-7 IC_50_ = 15.08 µM; 2′,4′-*O*-diacetyl-3-*O*-α-L-arabinopyranosyl-23-hydroxyolea-12-en-28-oic acid, A549 IC_50_ = 6.67 µM; H157 IC_50_ = 9.57 µM; HepG2 IC_50_ = 16.23 µM; MCF-7 IC_50_ = 15.23 µM; 3*β*-*O*-trans-feruloyl-2*α*-hydroxy-urs-12-en-28-oic acid, A549 IC_50_ = 12.8 µM; H157 IC_50_ = 5.66 µM; HepG2 IC_50_ = 9.5 µM; MCF-7 IC_50_ = 9.36 µM; leontoside A, A549 IC_50_ = 35.24 µM; H157 IC_50_ = 36.45 µM; HepG2 IC_50_ = 46.4 µM; MCF-7 IC_50_ = 52.06 µM; 4′-*O*-acetyl-3-*O*-*α*-l-arabinopyranosyl-23-hydroxyolea-12-en-28-oic acid, A549 IC_50_ = 22.94 µM; H157 IC_50_ = 21.21 µM; HepG2 IC_50_ = 27.13 µM; MCF-7 IC_50_ = 26.72 µM; 3′-*O*-acetyl-3-*O*-*α*-l-arabinopyranosyl-23-hydroxyolea-12-en-28-oic acid, A549 IC_50_ = 34.18 µM; H157 IC_50_ = 30.02 µM; HepG2 IC_50_ = 34.35 µM; MCF-7 IC_50_ = 37.32 µM; 2′-*O*-acetyl-3-*O*-*α*-l-arabinopyranosyl-23-hydroxyolea-12-en-28-oic acid, A549 IC_50_ = 33.14 µM; H157 IC_50_ = 27.54 µM; HepG2 IC_50_ = 34.5 µM; MCF-7 IC_50_ = 35.66 µM	Doxorubicin, A549 IC_50_ = 1.68 µM; H157 IC_50_ = 0.85 µM; HepG2 IC_50_ = 1.57 µM; MCF-7 IC_50_ = 0.9 µM	[12]
	Roots	3-*O*-[*β*-d-xylopyranosyl-(1→4)-*β*-d-glucopyranosyl-(1→4)][α-l-rhamnopyranosyl-(1→3)]-*β*-d-glucopyranosyl-(1→3)-α-l-rhamnopyranosyl-(1→2)-α-l-arabinopyranosylhederagenin	Lung A549, liver H157 and HepG2, and breast MCF-7 cell lines	SRB method, cytotoxicity (48 h)	A549 IC_50_ = 6.94 µM; H157 IC_50_ = 9.06 µM	-	[96]
*D. asperoides*	Roots	Water extract	Human mammary carcinoma derived triple negative MDA-MB-231 cells	Cell Titre Glo 2.0 assay, cell viability (7 d); flow cytometer (cell cycle); Western Blot; caspase 3/7 activity	Cell viability IC_50_ = 15 µg/mL; arrest cycle growth of cells in G_2_/M phase; induction of apoptosis by increasing pro-apoptotic caspase 3/7 activity and inhibiting the expression of BRAF, p-ERK, MEK, pPI3K, pAKT, and cyclin-dependent kinase 4/6 in a dose-dependent manner	-	[175]
	Roots	Akebia saponin PA	Human gastric cancer AGS, MKN-45, SNU-638, and KATO III cell lines	MTT assay, cell viability (24 h); Annexing V/propidium (PI) staining; Western Blot	Cell viability AGS IC_50_ = 30.3 µM; cell viability MKN-45 IC_50_ = 24.1 µM; cell viability SNU-638 IC_50_ = 27.6 µM; cell viability KATO III IC_50_ = 36.5 µM; an increase in apoptotic cells (AGS) to 9.46%, 19.33%, and 48.20% at a concentration of 20 µM, 30 µM, and 40 µM, respectively; an increase in AGS cell number in the sub-G_1_ phase; activation of caspase-3, cleavage of PARP-1, mitogen-activated protein kinases (MAPKs), and p38/c-Jun N-terminal kinase (JNK); induction of autophagy through PI3K/AKT/mTOR and AMPK/mTOR pathways	-	[173]
	Roots	Water-soluble polysaccharide (ADAPW) with the molecular weight of 16 kDa	Human osteosarcoma cell line HOS cells	MTT assay, cell viability (24 h); Annexin V-FITC/PI staining; Western Blot	Inhibition of cell growth (at the concentration of 400 µg/mL, the cell survival rate ˂35%); induction of apoptosis in a concentration-dependent pattern (at a concentration of 100–400 µg/mL, the number of apoptotic cell increase to 23.7%-55.3%); down-regulation of PI3K and pAkt protein level; reduction of mitochondrial membrane potential; an increase in intracellular ROS level		[11]
*D. fullonum*	Leaves	Iridoid glycosides fraction of 80% methanol extract	Human breast cancer cell lines MCF7 and MDB-MB-231 and human cervical cancer cell line HeLa	WST-1 assay, cell viability (72 h)	Decrease in cell survival; the viability of cells 64%, 69.5%, and 78.9% for MCF7, MDB-MB-231, and HeLa, respectively	-	[174]
	Leaves and flowers	96% ethanol extract	Human hepatocellular carcinoma cell lines Hep3B, HepG2, PLC/PFR/5, and SNU-182	ATPlite assay, cell viability (8 h)	Hep3B IC_50_ > 100 µg/mL; HepG2, PLC PFR5, SNU-182 antiproliferative effect not detected	-	[176]
*D. japonicus*	Roots	Saponin XII	acute myeloid leukemia OCI-AML3 cells	Cell viability (24 h) and cell cycle progression were analyzed by flow cytometry to determine the DNA content of cell nuclei stained with propidium iodide (PI)	Apoptosis cell death at a concentration of 1–2 µg/mL (0.648–1.295 µM); an increase in the number of cells in the G_0_/G_1_ phase of the cell cycle and a decrease in the number of cells in the S and G_2_/M phases, and activation of caspase-3	-	[40]
*S. atropurpurea* subsp. *maritima*	Leaves	Methanolic extract	Human epithelial colorectal adenocarcinoma Caco-2	MTT assay, cell viability (48 h); Annexin-V/PI Double-Staining analysis of apoptotic cells, flow cytometer; Multicaspase assay; quantitative reverse transcription Real-Time PCR (RT-qPCR)	Plant extract at a concentration of IC_10_, IC_20_, or IC_30_ enhances the toxicity of doxorubicin; cell viability IC_50_ = 1.04 µg/mL; the combination of doxorubicin with plant extract at a concentration of IC_50_ and IC_10_ results in an increase in the percentage of apoptotic cells, the percentage of caspase-activated cells, mRNA levels of the apoptosis related-genes (Bax, caspase-3, p21), and a decrease in expression level of anti-apoptotic genes (Bcl-2)	doxorubicin cell viability IC_50_ = 2.41 µg/mL	[81]
	Fruits	Water extract, silver nanoparticles	Human multiple myeloma U266 cell line and human breast cancer cell line MDA-MB-231	MTT assay, cell viability (48 h)	U266 IC_50_ = 10 µg/mL; MDA-MB-231 IC_50_ = 12 µg/mL	-	[56]
*S. stellata*	Whole plants	Scabiostellatosides A-H	Fibrosarcoma HT1080 cell line	WST1 assay, cell viability (72 h)	Scabiostellatoside B, IC_50_ = 49 µM; scabiostellatoside D, IC_50_ = 40 µM; scabiostellatoside E, IC_50_ = 38 µM; scabiostellatoside F, IC_50_ =12 µM; scabiostellatoside H, IC_50_ = 40 µM; scabiostellatoside A, scabiostellatoside C, scabiostellatoside G not cytotoxic at a concentration of 50 µM	Doxorubicin, IC_50_ = 0.59 µM	[48]
	Whole plants	Iridoids (7-*O*-(*E*-caffeoyl)-sylvestroside I and 7-*O*-(*E*-*p*-coumaroyl)-sylvestroside I	Fibrosarcoma HT1080 cell line	WST1 assay, cell viability (72 h)	7-*O*-(*E*-caffeoyl)-sylvestroside I, IC_50_ = 35.9 µg/mL; 7-*O*-(*E*-*p*-coumaroyl)-sylvestroside I, IC_50_ > 100 µg/mL	-	[75]

## Data Availability

Not applicable.

## Supplementary Materials

The following supporting information can be downloaded at: https://www.mdpi.com/article/10.3390/molecules28093754/s1, Table S1. The list of accepted species in the genera *Dipsacus* and *Scabiosa* according to Plants of the World Online database [9].
