# Supplementary material for: Dipsacus and Scabiosa Species—The Source of Specialized Metabolites with High Biological Relevance: A Review
Source: Molecules. 2023 Apr 27;28(9):3754. doi: 10.3390/molecules28093754 (PMC10180103; doi:10.3390/molecules28093754)
Supplement: Supplementary file 1 [file molecules-28-03754-s001.zip › molecules-2294302-supplementary.pdf]

Table S1. The list of accepted species in the genera *Dipsacus* and *Scabiosa* according to Plants of the World Online database [9].

Accepted species name		
Dipsacus spp.	*Scabiosa* spp.	
D. asper Wall. ex DC.	*S. adzharica* Schchian	*S. japonica* Miq.
D. atratus Hook.f. & Thomson ex C.B.Clarke	*S. africana* L.	*S. jezoensis* Nakai
D. atropurpureus C.Y.Cheng & Z.T.Yin	*S. albanensis* R.A.Dyer	*S. lacerifolia* Hayata
D. azureus Schrenk ex Fisch. & C.A.Mey.	*S. amoena* J.Jacq.	*S. lachnophylla* Kitag.
D. cephalarioides V.A.Matthews & Kupicha	*S. andryalifolia* (Pau) Devesa	*S. libyca* Alavi
D. chinensis Batalin	*S. angustiloba* (Sond.) B.L.Burtt ex Hutch.	*S. lucida* Vill.
D. comosus Hoffmanns. & Link	*S. arenaria* Forssk.	*Scabiosa* × *lucidula* Beck
D. ferox Loisel.	*S. atropurpurea* L.	*S. mollissima* Viv.
D. fullonum L.	*S. austroafricana* Heine	*S. nitens* Roem. & Schult.
D. gmelinii M.Bieb.	*S. balcanica* (Velen.) Velen.	*S. ochroleuca* L.
D. inermis Wall.	*Scabiosa* × *beauverdiana* Palez.	*S. owerinii* Boiss.
D. japonicus Miq.	*S. bipinnata* K.Koch	*S. paphlagonica* Bornm.
D. laciniatus L.	*S. buekiana* Eckl. & Zeyh.	*S. parielii* Maire
D. leschenaultii Coult. ex DC.	*S. canescens* Waldst. & Kit.	*S. parviflora* Desf.

D. narcisseanus Lawalrée	*S. cartenniana* A.Pons & Quézel	*S. praemontana* Privalova
D. pilosus L.	*S. cephalarioides* Lojac.	*S. pyrenaica* All.
D. pinnatifidus Steud. ex A.Rich.	*S. cinerea* Lapeyr. ex Lam.	*S. semipapposa* Salzm. ex DC.
Dipsacus × *pseudosylvestris* Schur	*S. colchica* Steven	*S. silenifolia* Waldst. & Kit.
D. sativus (L.) Honck.	*S. columbaria* L.	*S. sirnakia* Yıld.
D. strigosus Willd. ex Roem. & Schult.	*S. comosa* Fisch. ex Roem. & Schult.	*S. sivrihisarica* Yıld.
D. valsecchii Camarda	*S. correvoniana* Sommier & Levier	*S. solymica* (Parolly, Eren & Nordt) Göktürk
D. walkeri Arn.	*S. corsica* (Litard.) Gamisans	*S. sosnowskyi* Sulak.
	S. crinita Kotschy & Boiss.	*S. taygetea* Boiss. & Heldr.
	S. daucoides Desf.	*S. tenuis* Spruner ex Boiss.
	S. drakensbergensis B.L.Burtt	*S. thysdrusiana* Le Houér.
	S. eremophila Boiss.	*S. transvaalensis* S.Moore
	S. farinosa Coss.	*S. triandra* L.
	S. fumarioides Vis. & Pančić	*S. triniifolia* Friv.
	S. galianoi Devesa, Ortega Oliv. & J.López	*S. turolensis* Pau
	S. holosericea Bertol.	*S. tuzluca* Yıld.
	S. hyrcanica Steven	

	S. imeretica (Sommier & Levier) Sulak.<br>*S. incisa* Mill.<br>*S. ispartaca* Yıld.	*S. tysonii* L.Bolus<br>*S. velenovskiana* Bobrov<br>*S. vestina* Facchini ex W.D.J.Koch<br>*S. webbiana* D.Don

Family Caprifoliaceae includes six subfamilies (Diervilloideae, Caprifolioideae, Linnaeoideae, Morinoideae, Dipsacoideae, Valerianoideae) and one genus *Zabelia* [11].
